# Surface Engineering of Methylammonium Lead Bromide Perovskite Crystals for Enhanced X-ray Detection

**DOI:** 10.1021/acs.jpclett.3c02061

**Published:** 2023-10-05

**Authors:** Abraha Tadese Gidey, Yuki Haruta, Artur P. Herman, Miłosz Grodzicki, Anna M. Melnychenko, Dominika Majchrzak, Somnath Mahato, Ernest Rogowicz, Marcin Syperek, Robert Kudrawiec, Makhsud I. Saidaminov, Ahmed L. Abdelhady

**Affiliations:** † ŁUKASIEWICZ Research Network PORT-Polish Center for Technology Development, 54-066 Wrocław, Poland; ‡ Department of Chemistry, University of Victoria, 3800 Finnerty Road, Victoria, British Columbia V8P 5C2, Canada; § Department of Semiconductor Materials Engineering, Faculty of Fundamental Problems of Technology, Wrocław University of Science and Technology, Wybrzeże Wyspiańskiego 27, 50-370 Wrocław, Poland; ∥ Department of Experimental Physics, Wrocław University of Science and Technology, Wybrzeże Wyspiańskiego 27, 50-370 Wrocław, Poland; ⊥ Department of Electrical & Computer Engineering, University of Victoria, 3800 Finnerty Road, Victoria, British Columbia V8P 5C2, Canada; # Centre for Advanced Materials and Related Technologies (CAMTEC), University of Victoria, 3800 Finnerty Road, Victoria, British Columbia V8P 5C2, Canada; ¶ Department of Chemistry, Khalifa University, P.O. Box 127788, Abu Dhabi, United Arab Emirates; ■ Advanced Materials Chemistry Center (AMCC), Khalifa University, P.O. Box 127788, Abu Dhabi, United Arab Emirates

## Abstract

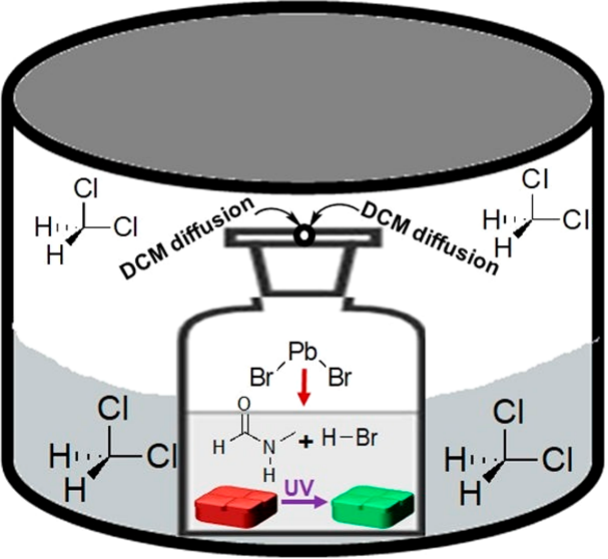

The surface quality of lead halide perovskite crystals can extremely influence their optoelectronic properties and device performance. Here, we report a surface engineering crystallization technique in which we in situ grow a polycrystalline methylammonium lead tribromide (MAPbBr_3_) film on top of bulk mm-sized single crystals. Such MAPbBr_3_ crystals with a MAPbBr_3_ passivating film display intense green emission under UV light. X-ray photoelectron spectroscopy demonstrates that these crystals with emissive surfaces are compositionally different from typical MAPbBr_3_ crystals that show no emission under UV light. Time-resolved photoluminescence and electrical measurements indicate that the MAPbBr_3_ film/MAPbBr_3_ crystals possess less surface defects compared to the bare MAPbBr_3_ crystals. Therefore, X-ray detectors fabricated using the surface-engineered MAPbBr_3_ crystals provide an almost 5 times improved sensitivity to X-rays and a more stable baseline drift with respect to the typical MAPbBr_3_ crystals.

The growth of high-quality lead halide perovskite (LHPs) crystals is crucial to fully understand their optoelectronic properties.^1−3^ Various approaches have been used to grow MAPbX_3_ (MA = methylammonium and X = Cl, Br, or I) single crystals including inverse temperature crystallization (ITC),^4^ antisolvent vapor-assisted crystallization (AVC),^5^ bottom-seeded solution growth (BSSG),^6^ top-seeded solution growth (TSSG),^7^ solvent acidolysis crystallization (SAC),^8^ and low temperature-gradient crystallization.^9^ These various crystallization techniques can result in crystals with different optoelectronic properties. For instance, Ding et al.^10^ have demonstrated that MAPbI_3_ crystals grown by the ITC method would possess higher photoluminescence (PL) intensity and longer charge carrier PL decay time compared to the crystals obtained by the BSSG method. Interestingly, this recorded PL behavior is despite the higher crystalline quality of the BSSG grown crystals as suggested by X-ray diffraction (XRD) rocking curve measurement.^11^ Furthermore, it has been previously demonstrated that the surface quality of the bulk LHP crystals can highly affect their optoelectronic properties and hence their device performance.^12−14^ In particular, surface defects, which result in nonradiative charge carrier recombination pathways, were reported as the major concern for the utilization of HP-based single crystals in optoelectronic devices.^15−19^

In order to modify surface quality of bulk LHP crystals, several works sought after surface passivation through the introduction of an additive in the precursor solution or by post-treatment of the grown crystals with passivating solution.^20−25^ For instance, Liu et. al^24^ introduced coordinating organic molecules in perovskite precursor solution to regulate the growth of perovskite single crystals impacting both nucleation and crystal growth processes and hence improving their optoelectronic properties compared to crystals grown without the additive. However, several of the potential passivating agents are nonconducting organic molecules, which impact the device performance negatively. Alternatively, other works investigated growing LHP/LHP heterostructures (MAPbCl_3_–MAPbBr_3_ or MAPbI_3_–MAPbBr_3_) for surface passivation.^26,27^ A heterostructure formed by depositing FAPbI_3_ (FA = formamidinium) thin film on MAPb(Cl/Br)_3_ single crystal was found efficient for strain engineering and hence for enhanced photodetector performance.^28^ Nevertheless, these heterostructures could suffer from ion exchange between the two layers, as the halide ion penetration in LHP single crystals can reach the millimeter range.^29^

LHP bulk crystals have been explored for X-ray detection with a focus on the MAPbBr_3_ composition because of the MAPbBr_3_ crystals’ high stopping power, high charge collection efficiency, and high X-ray sensitivity.^30,31^ To further enhance the X-ray detection capabilities, surface passivation techniques were investigated. One approach is through utilizing a heterostructure combining three-dimensional (3D) LHP crystals with a 2D perovskite layer, in which the 2D layer is deposited on top of the 3D LHP crystals.^32^

In this work, we grow MAPbBr_3_ crystals at room temperature using a modified SAC technique resulting in crystals with intense green surface emission under UV light, which is unusual in such bulk mm-sized MAPbBr_3_ crystals. This crystallization process allows the in situ deposition of a MAPbBr_3_ thin film on the MAPbBr_3_ crystals, thus explaining the intense green emission. Unlike the typical MAPbBr_3_ crystals that do not show green emission under UV light, the surfaces of the emissive crystals we develop here are Br-rich but N-deficient. Time-resolved photoluminescence measurements and electrical measurements indicate that the MAPbBr_3_ film passivation on the MAPbBr_3_ crystals leads to decreased surface defects. The MAPbBr_3_ crystals grown by the modified SAC technique offer improved sensitivity to X-rays (549.9 μC Gy_air_^–1^ cm^–2^, which is 4.8 times higher) and the more stable baseline drift (4.8 × 10^–4^ nA cm^–1^ V^–1^ s^–1^) compared to the control device based on the MAPbBr_3_ crystals grown without any modification.

The crystals were grown using solvent acidolysis crystallization (SAC), in which *N*-methylformamide (NMF) is used as both solvent and source of methylammonium (MA) cations.^8,33−36^ The control MAPbBr_3_ crystals (labeled as Control-1M), synthesized from a 1 M PbBr_2_ solution in NMF and HBr without the use of any antisolvent, were found to be nonemissive under UV light (Figure 1a–b), which is expected from such bulk mm-sized crystals. On the other hand, the crystals developed through slow diffusion of an antisolvent (DCM) to a 0.2 M PbBr_2_ solution in NMF and HBr (labeled as DCM-0.2M) resulted in the formation of crystals with bright green emission under UV light (Figure 1c–d). Figure 1e–f represents the optical microscopy images of Control-1M, while the DCM-0.2M surface images are shown in Figure 1g–h. Both the top and bottom surfaces of the DCM-0.2M crystals appear to be rougher, with respect to the Control-1M crystals, which is in line with the scanning electron microscopy (SEM) images shown in Figure 1i–l. Figure S1a–c, in the Supporting Information, represents atomic force microscopy (AFM) results of the Control-1M bottom face, while Figure S1d–f shows the corresponding images for the DCM-0.2M. The AFM analysis was performed in different places on the sample surface as well as with different scan sizes. Comparing a 5 × 5 μm^2^ scan for both samples (Figure S1a,d), the higher surface root-mean-square (RMS) roughness was observed in the case of DCM-0.2M . The bottom face of the Control-1M MAPbBr_3_ exhibits step-like areas with RMS equal to 2.1 nm. In the case of the DCM-0.2M, a terrace-like surface is also observed, however with an RMS equal to 7.6 nm. Figure S1b,1e shows smaller size AFM images measured in other regions than the one presented in Figure S1a,d, for Control-1M and DCM-0.2M, respectively. For these images, the line scan profile was extracted, which allowed for detailed roughness analysis. In Figure S1c, representing the line scan profile for the Control-1M MAPbBr_3_, steps with height up to ∼10 nm can be observed. The line scan profile for DCM-0.2M, presented in Figure S1f, shows terraces with a height up to ∼40 nm. These AFM results concluded that the DCM-0.2M crystals exhibit higher surface roughness with respect to the Control-1M.

**Figure 1:**
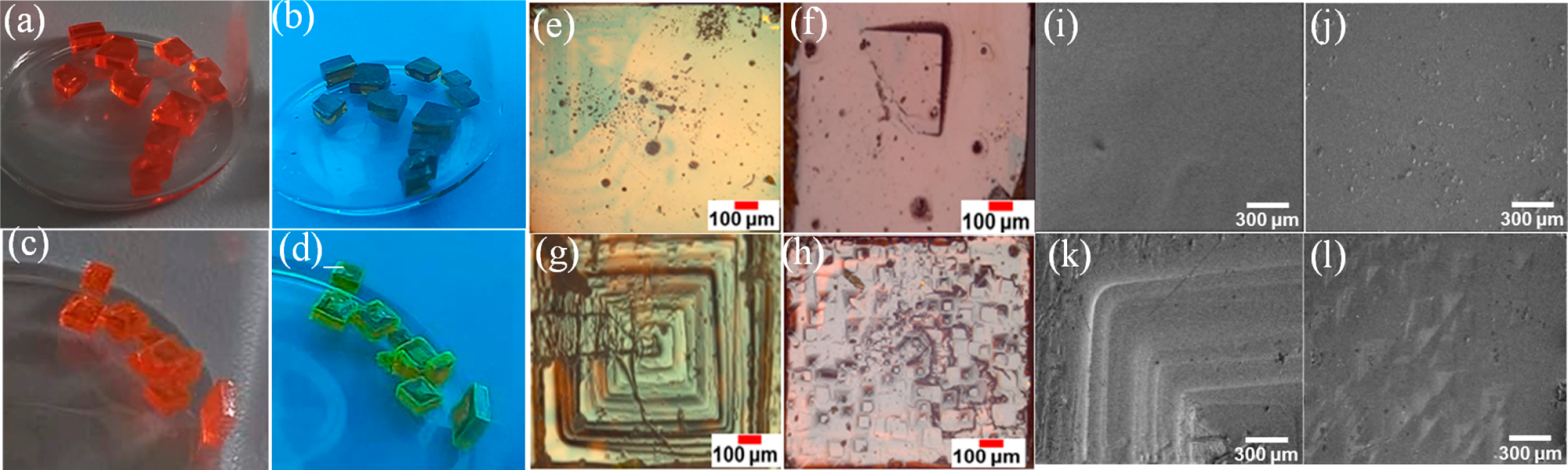
(a–b) Photographs of the Control-1M MAPbBr_3_ crystals under normal light and under UV light, respectively. (c–d) Corresponding photographs of DCM-0.2M, respectively. (e–f) Optical microscopy images of top and bottom faces of the Control-1M MAPbBr_3_ crystals, respectively. (g–h) Corresponding optical microscopy images of DCM-0.2M crystals top and bottom faces, respectively. (i–j) SEM images of top and bottom faces of the Control-1M MAPbBr_3_ crystals, respectively. (k–l) Corresponding SEM images of DCM-0.2M crystals top and bottom faces, respectively.

The presence of some microstructures on the surface of halide perovskite crystals, as in our DCM-0.2M crystals, was previously observed by different groups.^37−39^ While such structures have been associated with how the crystals were removed from the growth solution and how they were dried afterward,^37^ this can be excluded here as both types of crystals (Control-1M and DCM-0.2M) were handled identically. In our DCM-0.2M crystals, it appears that the microstructures explicitly observed on the bottom surface (Figure 1h, part l) correspond to a polycrystalline layer growing on the crystal. This can be more clearly seen when focusing on the edges of the crystal in the optical microscopy photos represented in Figure S2a. The same observation is also recorded when we examined the side faces of the DCM-0.2M crystals (Figure S2b). Consequently, the DCM-0.2M crystals can be described as MAPbBr_3_ film/MAPbBr_3_ crystal, while the Control-1M crystals can be seen simply as bare MAPbBr_3_ crystals. The deposition of the polycrystalline layer in the case of DCM-0.2M crystals is probably due to the manipulation of the precursor solution, as it is well-documented that even minor variations in the halide perovskite precursor concentration and/or composition can lead to significant changes in the quality of the formed material.^33,40,41^

Figure S3 shows the X-ray diffraction (XRD) patterns of the ground crystals of both samples (Control-1M and DCM-0.2M). The XRD patterns agree with the previously reported cubic perovskite structure of MAPbBr_3_ (ICSD code 252415).^42,43^ No impurity peaks were observed in the XRD result, which implied that pure crystals are grown from both methods.

The X-ray photoelectron spectroscopy (XPS) spectra acquired from both Control-1M and DCM-0.2M crystals are shown in Figures S4 and S5. The binding energies obtained for our crystals are in line with other results previously published for MAPbBr_3_ perovskite.^44−46^ However, the surfaces of both crystals were significantly different in terms of the elemental ratio. The Control-1M crystals show ratios of 2.62 and 0.98 for Br/Pb and N/Pb, respectively, while DCM-0.2M indicates ratios of 3.15 and 0.72, respectively. The results for the Control-1M are in accordance with previous reports in which MAPbBr_3_ crystals were reported as halide-deficient,^14,46^ which can lead to defects due to the halide vacancies that can act as nonradiative recombination centers.^47,48^ On the other hand, the DCM-0.2M is slightly halide-rich but MA-deficient, which is another source of defect leading to increased quenching of the charge carriers.^49^ Nevertheless, the activation energy of the MA vacancies is much higher than their corresponding values for halide vacancies, and hence, MA vacancies are much less prone to migration, which is beneficial for optoelectronic devices.^50,51^ Some previous reports revealed that halide- or MA-deficient perovskites are associated with the presence of metallic Pb,^44,52^ which is not the case in our present study—both Control-1M and DCM-0.2M crystals are free of Pb metallic.

Figure S6 shows the shallow core level of Br 4s at 21.2 eV and Pb 5d at 19.1 eV for Control-1M. The peaks are located 0.2 eV deeper for the DCM-0.2M. A work function (*W*_F_) of 5.0 eV for both samples is calculated from the difference in photon energy (1486.6 eV) and the cutoff energy.^45^ On the other hand, the valence band maximum (VBM) lies at 1.5 and 2.0 eV below the Fermi level for Control-1M and DCM-0.2M, respectively (Figure S5). These values allow us to determine an ionization energy (*I*_E_) from the relationship *I*_E_ = *W*_F_ + VBM. This gives *I*_E_ of 6.5 and 7.0 eV for the Control-1M and DCM-0.2M, respectively. The *W*_F_ and the *I*_E_ value of 6.5 eV are in line with previously reported results.^53,54^ The *I*_E_ value of 7.0 eV, considering an identical bandgap for both crystals, indicates that the DCM-0.2M is more n-type with respect to the Control-1M. The difference can be attributed to the difference in the surface stoichiometry of both samples.^44^

To compare the quality of the two samples (Control-1M and DCM-0.2M), time-resolved photoluminescence (TRPL) was measured at the same excitation conditions at room temperature. Figure 2a,b shows streak camera images for Control-1M and DCM-0.2M, respectively, together with reflectance (R) and time-integrated PL spectra. The peak-like feature observed in the R spectrum for both samples close to 2.3 eV (see horizontal cyan dashed lines) is attributed to the free exciton (FX) and band-to-band (b-b) absorption. The excitonic contribution cannot be neglected, as the exciton binding energy (25–40 meV)^55^ is higher than the *kT* energy at room temperature (26 meV). Two Gaussian peaks fit PL spectra, and one of them, which is observed at higher energy, can be attributed to the mentioned FX/b-b emission (i.e., both emissions are between extended states). The broad PL peak, which dominates in the spectrum below the bandgap (as determined by R measurements), is attributed to emission through localized states (also called defect-assisted emission (DAE)). The character of this emission can be both excitonic and nonexcitonic because of comparable values of the exciton binding energy and thermal energy at room temperature. Point defects or other crystal imperfections can localize excitons and carriers, which recombine radiatively or nonradiatively.^56^ The nonradiative recombination leads to shortening of PL decay time of the free exciton and band-to-band recombination. This is strongly manifested when measurements are performed at low excitation conditions, and therefore, TRPL was measured at the average power density of ∼20 W/cm^2^, which can be considered relatively low.

**Figure 2:**
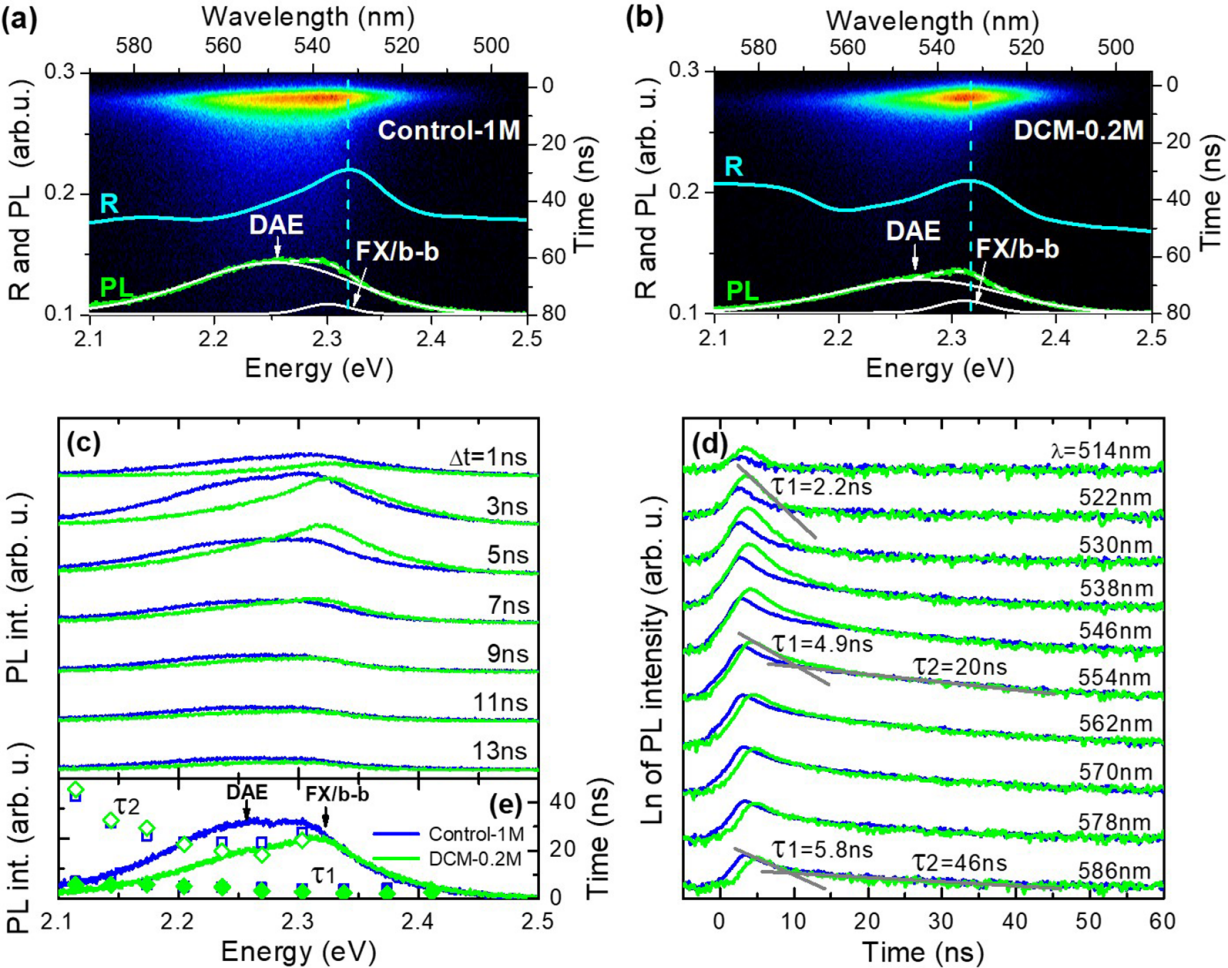
Streak camera images of the time-resolved photoluminescence (TRPL) of Control-1M (a) and DCM-0.2M (b) recorded at room temperature together with reflectance (R) (cyan color curves) and PL spectra integrated over time (green lines) and their decomposition for two Gaussian peaks (white lines). (c) PL spectra for different time delays (Δ*t*) after excitation. (d) Decay of PL at selected wavelengths. (e) Time-integrated PL spectra and decay times (τ_1_ and τ_2_) extracted from the PL decay curves.

The direct comparison of PL spectra for different time delays (Δ*t*) after excitation and decay of PL at selected wavelengths is shown in Figure 2c,d, respectively. Apparent differences in the shape of the PL spectra can be observed in Figure 2c. They are attributed to the different quality of the investigated samples, i.e., Control-1M has stronger defect-assisted emission due to the higher concentration of point defects or other imperfections. The PL decay curves vary with the wavelength; see Figure 2d. At shorter wavelengths, these curves can be described as time constant in the range of 2–3 ns. This is the free exciton and/or free carrier recombination, in which dynamics are shortened by exciton (carrier) capture by localized states. The intrinsic exciton (carrier) lifetime in MAPbBr_3_ is expected to be longer than the PL decay time observed for the recombination between extended states, i.e., the high-energy emission peak.

In the transparency region (i.e., the region below the energy gap determined by reflectance), the PL decay curves cannot be described by a single exponent, and therefore, two time constants (τ_1_ and τ_2_) were determined for curves recorded for wavelengths longer than 530 nm. The τ_1_ increases from ∼4 to ∼6 ns, and τ_2_ increases from ∼20 to ∼46 ns going to 586 nm. The observed differences in time constant for Control-1M and DCM-0.2M are rather insignificant; see Figure 2e. Such an elongation of PL decay time with emission wavelength is a fingerprint of localized emission^57,58^ and strongly supports the interpretation of this PL band as an emission of localized excitons and carriers. The contribution of this emission to the total emission is larger for Control-1M, and therefore, this sample may be treated as a lower quality when compared to the DCM-0.2M. In general, the value of PL decay time (*τ*_PL_) can be treated as an indicator of sample quality: the longest time constant is correlated to the higher sample quality due to the lower contribution of nonradiative recombination according to eq 1
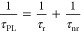
1where *τ*_r_ is the intrinsic radiative lifetime and *τ*_nr_ is the nonradiative time, and the 1/*τ*_nr_ rate increases with the number of nonradiative centers. However, this is the case of free exciton or free carrier recombination. In the case of localized emission, the direct comparison of the PL decay time is not trivial due to the saturation of defect states that is altered with the excitation power. So far, longer PL decay constants were reported for MAPbBr_3_, but they were obtained at higher excitation conditions.^59,60^ In our case, much longer PL decay times were obtained for localized emission for Control-1M and DCM-0.2M at the excitation by ps laser with the average power density of ∼1.5 W/cm^2^; see Figure S7 in Supporting Information. Again, it is observed that the PL decay time is longer at longer wavelengths, which confirms the localized nature of the emission below the energy gap.

The trap densities of both crystals were measured by using four crystals of each with the pulsed-voltage space-charge limited current (PV-SCLC) method (Figures S8 and S9) that has been proposed to properly evaluate perovskite crystals while eliminating the well-known ion migration effect during the measurement.^61^ As shown in Figure S10, both crystals showed similar trap densities (DCM-0.2M: 11.5 × 10^9^ cm^–3^, Control-1M: 9.0 × 10^9^ cm^–3^ on average). The trap density obtained by PV-SCLC reflects the bulk property rather than the surface property. On the other hand, when we applied continuous voltages to the devices, we observed less hysteresis in the DCM-0.2M device compared to the Control-1.0M (Figure S11), suggesting less charge accumulation at the interface between perovskite and electrodes, which indicates a better surface of the DCM-0.2M crystal. These results suggest that DCM-0.2M and Control-1M have similar trap densities in the bulk; however, DCM-0.2M has less defects at the surface.

Based on the Control-1M and DCM-0.2M crystals, X-ray detectors with the asymmetric device configuration of Au/MAPbBr_3_/Ga were fabricated, where the Au and Ga were chosen as metal electrodes with large and low work functions, respectively (Figure 3a). As shown in Figure 3b, both devices showed typical asymmetric dark *J*–*V* curves, suggesting that Schottky junctions were successfully formed. The Control-1M-based device showed almost 10 times higher leakage current at reversed bias compared to the DCM-0.2M-based device. We attribute this high leakage current to high defect densities at the surface of the Control-1M crystals, in agreement with the PL measurement.

**Figure 3:**
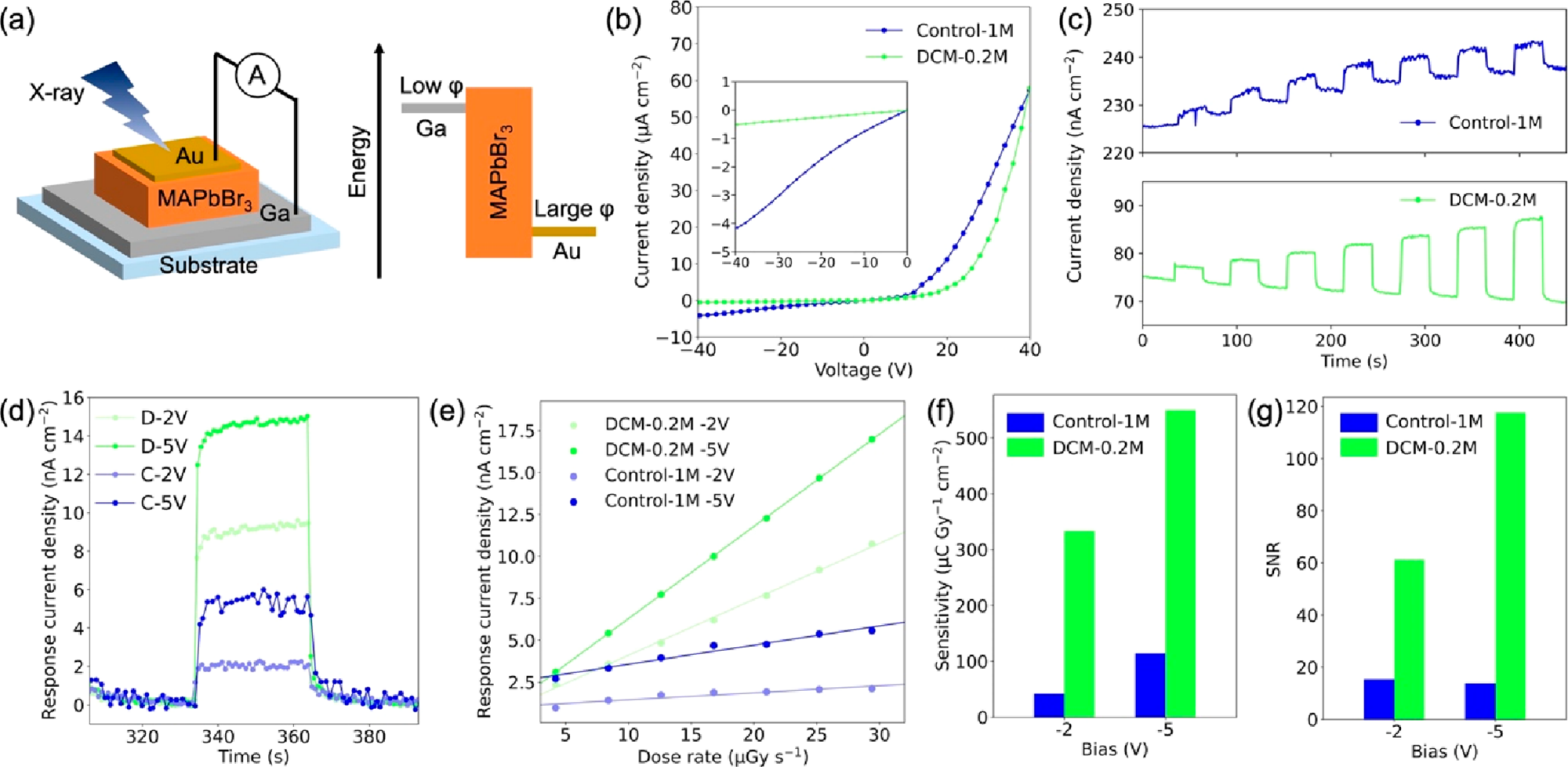
(a) Schematic of the device structure and its band diagram. (b) Dark current densities of the devices. (c) Response current densities of the devices to the various dose rates of X-rays at a bias of −5 V. (d) Net response current densities of the devices to 25.2 μGy_air_ s^–1^. (e) Relationship between the dose rates and the response current densities. (f) Sensitivities and (g) signal-to-noise ratios of the devices.

Figure 3c shows the X-ray response currents of the Control-1M- and DCM-0.2M-based devices at the bias of −5 V. The X-ray dose rate was controlled in the range of 4.2–29.4 μGy_air_ s^–1^ using a Comet MXR-160/22 X-ray tube with a tungsten anode as an X-ray source.^62^ All measurements were performed under a vacuum (<0.2 Torr) to eliminate air ionization currents. Both devices clearly responded to the X-rays, which is confirmed by the increase in the current densities. To analyze the X-ray detection properties, the baseline current densities were extracted by a fitting, and the net response current densities were calculated by subtracting the baseline from the raw data (Figure S12). We observed that the baseline current densities drifted slightly while applying the bias, which is probably due to ion migration phenomena in the perovskite crystals and charge accumulation at the interface. The baseline drift of the DCM-0.2M was 4.8 × 10^–4^ nA cm^–1^ V^–1^ s^–1^, which is lower than the Control-1M (8.4 × 10^–4^ nA cm^–1^ V^–1^ s^–1^) and the previously reported perovskite X-ray detectors (CsPbBr_3_: 1.49 × 10^–2^ nA cm^–1^ V^–1^ s^–1^, MAPbI_3_: 2.0 × 10^–3^ nA cm^–1^ V^–1^ s^–1^).^63,64^ As seen in the representative net response current densities (Figure 3d), the DCM-0.2M showed higher response currents and lower noise currents, indicating better X-ray detection properties than Control-1M. Both devices showed linear responses to the X-ray dose rate in the range of 4.2–29.4 μGy_air_ s^–1^ (Figure 3e). The DCM-0.2M device showed an SNR of 117.6 for 25.2 μGy_air_ s^–1^ and a sensitivity of 549.9 μC Gy_air_^–1^ cm^–2^ at the bias of −5 V, while the Control-1M device showed an SNR of 13.9 and a sensitivity of 114.4 μC Gy_air_^–1^ cm^–2^ (Figure 3f,g). This result reiterates the importance of surface engineering in improving the performance of X-ray detectors using perovskite crystals. The obtained sensitivity is compared with that of the reported perovskite X-ray detectors (Table Supporting Information). Our detectors showed a high sensitivity among them even though the electric field is not high.

We also measured the response current of the DCM-0.2M device at a higher bias of −20 V (Figure S13). Although the DCM-0.2M device showed higher response currents leading to the sensitivity of 898.8 μC Gy_air_^–1^ cm^–2^, the current was unstable, the same as the Control-1M at the bias of −5 V (Figure 3c). The SNR for 25.2 μGy_air_ s^–1^ X-ray was 36.2, which is less than the SNR of 117.6 when the bias was −5 V.

In conclusion, the solvent acidolysis crystallization technique is used to grow both typical MAPbBr_3_ crystals and surface-engineered crystals through modifying the precursor solution concentration along with using an antisolvent to initiate the crystallization. The surface-engineered MAPbBr_3_ crystals were found to be surface passivated with a MAPbBr_3_ film, which could be the origin of their intense green emission under UV light. These MAPbBr_3_ film/MAPbBr_3_ crystals possessed less surface defect density, and their sensitivity to X-ray detection was close to 5 times higher with respect to the bare MAPbBr_3_ crystals. Our results shed highlights on the importance of homostructures for enhanced optoelectronic performance.

## Experimental Section

### Materials

Lead(II) bromide (PbBr_2_, 99.999% trace metals basis, Sigma-Aldrich), hydrobromic acid (HBr, ACS reagent, 48 wt % in H_2_O, ≥99.99%, Sigma-Aldrich), *N*-methylformamide (NMF, 99%, Sigma-Aldrich), and dichloromethane (DCM, ACS reagent, ≥99.5%, Sigma-Aldrich) were purchased and used without any further purification.

### Crystallization of MAPbBr_3_ Crystals

Methylammonium lead bromide (MAPbBr_3_) crystals were synthesized using modified solvent acidolysis crystallization (SAC).^8^ In brief, 0.2 M PbBr_2_ was dissolved in a mixture of *N*-methylformamide (NMF) and HBr (5.7:1 vol ratio). The solution was sonicated for 5 min and filtered through a 0.45 μm syringe filter. Then, the 10 mL solution was put in a small crystallization dish. The top of the crystallization dish was completely covered with aluminum foil except for a small hole made using a needle at the center of the dish. This dish was then placed in a larger crystallization dish containing 60 mL of DCM so that the antisolvent vapor could diffuse slowly into the precursor solution via the small hole to initiate the crystallization. Finally, the whole setup was kept undisturbed, and the crystallization took place from 48 to 72 h. Once crystals were isolated, they were dried over a ChemWipe and then in a vacuum oven at 40 °C overnight. For the control samples, the crystals were synthesized without antisolvent using 1.0 M PbBr_2_ in a mixture of NMF and HBr (5.7:1 vol ratio). The solution was sonicated for 20 min until all materials dissolved, and then, the solution was filtered through a 0.45 μm syringe filter. Then, 1 mL of this solution was placed in a 4 mL vial and kept undisturbed for 24 h. The crystals were collected and dried in the same manner as above.

### Crystal Characterization

*Atomic force microscopy* (AFM) measurements were performed on the bottom face of the crystals and were analyzed in noncontact mode and at room temperature using the ScientaOmicron UHV AFM system. *Optical microscopy* measurements were performed using a Keyence VHX-1000 Digital Microscope, while the surface morphology of the crystals was analyzed by *scanning electron microscopy* (SEM): Helios 450HP (Thermo Fisher Scientific, formerly FEl Company) with an Octane Pro EDS detector (EDAX Inc.). *X-ray diffraction* (XRD) spectra for the ground MAPbBr_3_ crystals were collected with the Empyrean X-ray diffractometer from Malvern Panalytical equipped with a PIXcel^3D^ detector using Cu Kα radiation (λ = 1.5406 Å). The crystalline phase in the samples was determined in X’Pert HighScore Plus program based on the best fit from the ICDD PDF4 + 2020 crystallographic base. *X-ray photoelectron spectroscopy* (XPS) analysis was carried out in an UHV system equipped with a hemispherical energy analyzer (Argus CU) and a monochromatic Al Kα radiation source (*hν* = 1486.6 eV). The photoelectrons were collected with a constant pass energy of 30 or 50 eV and an energy step of 0.1 or 1 eV, respectively, for core level line and survey scans. Electrons were acquired at takeoff angle of 90° to the surface, providing a maximum probing depth. The binding energies of spectra were referred to the Fermi level of analyzer, which was determined on a reference Au sample cleaned with Ar ions. No charging of samples was observed during measurements, eliminating the need for binding energy corrections in this study. The atomic ratios in the samples were evaluated based on regions defined for the N 1s, Pb 4f, and Br 3d peaks. CasaXPS software was used for the analysis. *Reflectance* measurements were performed by using the lock-in technique. For this purpose, a halogen lamp was used as a light source, a single-grating monochromator with a focal length of 0.5 m for light dispersion, and a silicon diode for signal detection. *Time-resolved photoluminescence* (TRPL) was measured with a standard PL setup at room temperature. As an excitation source, the Ti:sapphire oscillator with the second harmonic generation crystal and the pulse picker was used to provide ∼140 fs-long optical pulses with a photon energy *E*_exc_ = 2.76 eV (450 nm) and 250 kHz repetition rate. The average excitation power density was ∼20 W/cm^2^. The photoluminescence emission was filtered by a 0.3 m focal length monochromator, and the temporal evolution of the PL signal was detected by a streak camera. The effective time resolution of the system is approximately 20 ps. TRPL measurement in the ps excitation regime was performed using an experimental setup equipped with a monochromator (0.3 m focal length) and a time-correlated single photon counting module (Becker & Hickl SPC-150-NX with PMC-150-04 detector). Samples were excited using a picosecond pulsed laser (355 nm) operating at 1 MHz repetition rate and average power density of ∼1.5 W/cm^2^.

### Pulsed-Voltage Space-Charge Limited Current Measurements

The devices for PV-SCLC measurements were prepared by depositing carbon paste on each surface of the MAPbBr_3_ crystals.^65^ Then, pulsed voltages of 0.05–200 V were applied to the devices with the ON-time of 20 ms and the interval time of 120 s. Based on the log*I*–log*V* plots, we determined the onset voltage of the trap-filled limit regime (*V*_TFL_) as shown in Figures S8 and S9. The trap density (*n*_trap_) was calculated by using the following equation:
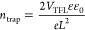
where ε_0_ represents the vacuum permittivity (8.85 × 10^–12^ F m^–1^), ε represents the relative dielectric constant of MAPbBr_3_ (25.5),^4^*e* represents the charge of the electron (1.6 × 10^–19^ C), and *L* represents the thickness of the crystal (mm).

### Device Fabrication and Characterization

The X-ray detectors with a device configuration of Au/MAPbBr_3_/Ga were fabricated as follows: Au electrodes (80 nm thick) were thermally evaporated on the surface of the as-deposited MAPbBr_3_ crystals. Then, Ga electrodes were deposited on the opposite surface by using liquified Ga and solidifying it at room temperature. The device area was 0.04 cm^2^.

For the X-ray measurement, a Comet MXR-160/22 X-ray tube with a tungsten anode was used as an X-ray source. The X-ray tube voltage was set to 40 kV. The dose rate was controlled by using a 0.36 mm thick Cu plate as an attenuator and changing the tube current (5–35 mA). The device was put in a homemade vacuum chamber with electrical feedthroughs and a Kapton window to let X-rays enter the chamber. The dose rate was calibrated by an ion chamber dosimeter. A Keithley 2450 sourcemeter was used for applying the bias and measuring the current. All measurements were performed under a vacuum (<0.2 Torr).

The dark current drift (*J*_drift_) was calculated as follows:
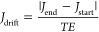
where *J*_start_ and *J*_end_ are the current densities at *t* = 0, 450 s, *T* is a total time of 450 s, and *E* is the applied electric field.

The net response current density (*J*_net_) was calculated by subtracting baseline currents from the response current. The X-ray sensitivity was calculated by extracting the slope of the linear fitting for the dose-rate-response current density plot. The signal-to-noise ratio (SNR) was calculated by *J*_net_/*J*_noise_ where the *J*_noise_ is defined as the standard deviation of the *J*_net_.

## Supporting Information Available

AFM measurements, optical microscopy images of DCM-0.2M MAPbBr_3_ crystals, and powder XRD patterns, XPS spectra, PL decay, log*I*–log*V* plots, *I*–*V* curves of the devices, and X-ray response of both crystals (PDF)
Transparent Peer Review report available (PDF)
