# Supplementary material for: Surface Engineering of Methylammonium Lead Bromide Perovskite Crystals for Enhanced X-ray Detection
Source: J Phys Chem Lett. 2023 Oct 5;14(40):9136–44. doi: 10.1021/acs.jpclett.3c02061 (PMC10577767; doi:10.1021/acs.jpclett.3c02061)
Supplement: Supplementary file 1 — jz3c02061_si_001.pdf [file jz3c02061_si_001.pdf]

Supporting Information

Surface Engineering of Methylammonium Lead Bromide Perovskite Crystals for Enhanced X-ray Detection

Abraha Tadese Gidey,[1,∥] *Yuki Haruta,*[2,∥] *Artur P. Herman,*[3] *Miłosz Grodzicki,*[1,3] *Anna M. Melnychenko,*[1,3] *Dominika Majchrzak,*[1] *Somnath Mahato,*[1] *Ernest Rogowicz,*[4] *Marcin Syperek,*[4] *Robert Kudrawiec,*[1,3,*] *Makhsud I. Saidaminov,*[2,5,6,*] *Ahmed L. Abdelhady*[7,8,1,*]

[1]*ŁUKASIEWICZ Research Network PORT-Polish Center for Technology Development, 54-066 Wrocław, Poland*

[2]*Department of Chemistry, University of Victoria, 3800 Finnerty Road, Victoria, British Columbia V8P 5C2, Canada*

[3]*Department of Semiconductor Materials Engineering, Faculty of Fundamental Problems of Technology, Wrocław University of Science and Technology, Wybrzeże Wyspiańskiego 27, Wrocław, Poland*

[4]*Department of Experimental Physics, Wrocław University of Science and Technology, Wybrzeże Wyspiańskiego 27, 50-370 Wrocław, Poland*

[5]*Department of Electrical & Computer Engineering, University of Victoria, 3800 Finnerty Road, Victoria, British Columbia V8P 5C2, Canada*

[6]*Centre for Advanced Materials and Related Technologies (CAMTEC), University of Victoria, 3800 Finnerty Road, Victoria, British Columbia V8P 5C2, Canada*

[7]*Department of Chemistry, Khalifa University, P.O. Box 127788, Abu Dhabi, United Arab Emirates*

[8]*Advanced Materials Chemistry Center (AMCC), Khalifa University, P.O. Box 127788, Abu Dhabi, United Arab Emirates*

[∥] These authors contributed equally

[*]Corresponding author(s): ahmed.abdelhady@ku.ac.ae, msaidaminov@uvic.ca, robert.kudrawiec@port.lukasiewicz.gov.pl

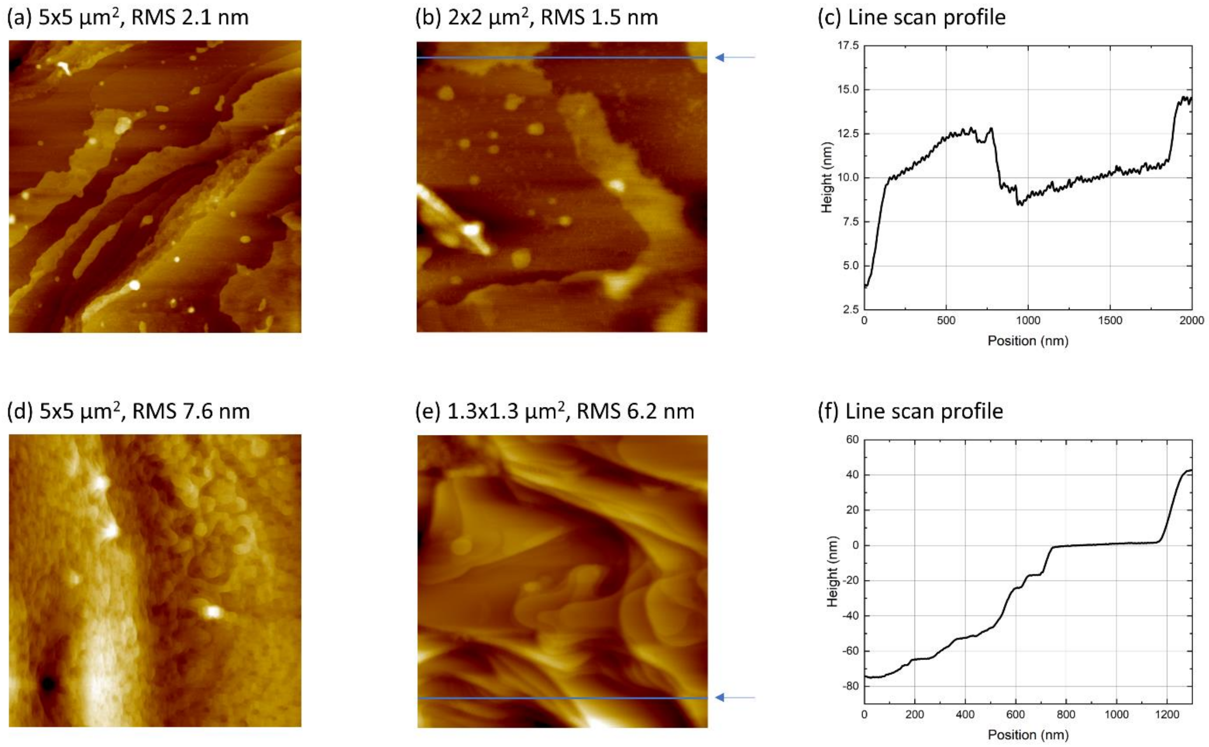

Figure S1. (a–c) AFM images with corresponding scan areas, roughness parameters and line scan profile of the Control-1M $MAPbBr_3$ crystal bottom face. (d–f) Corresponding AFM images of the DCM-0.2M crystal bottom face.

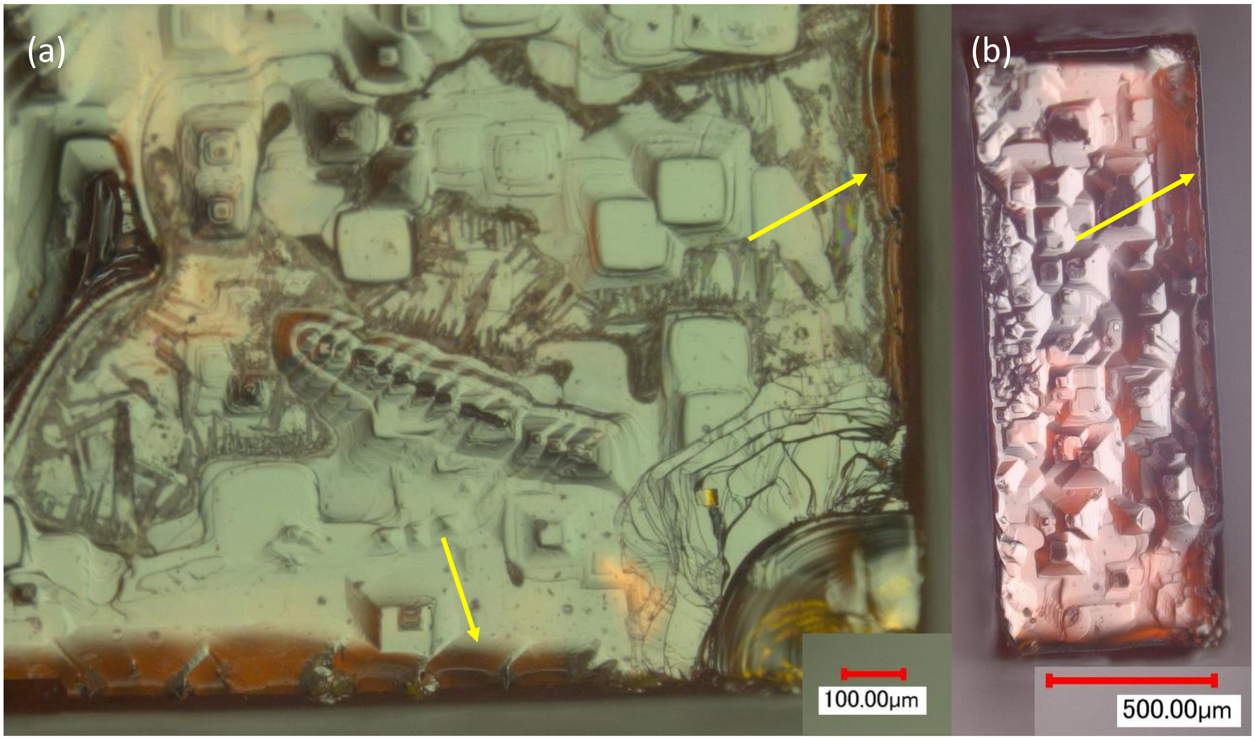

Figure S2. Photographs of (a) bottom face and (b) side face of the DCM-0.2M crystals with the yellow arrows pointing at locations suggesting there is a polycrystalline layer on the crystal.

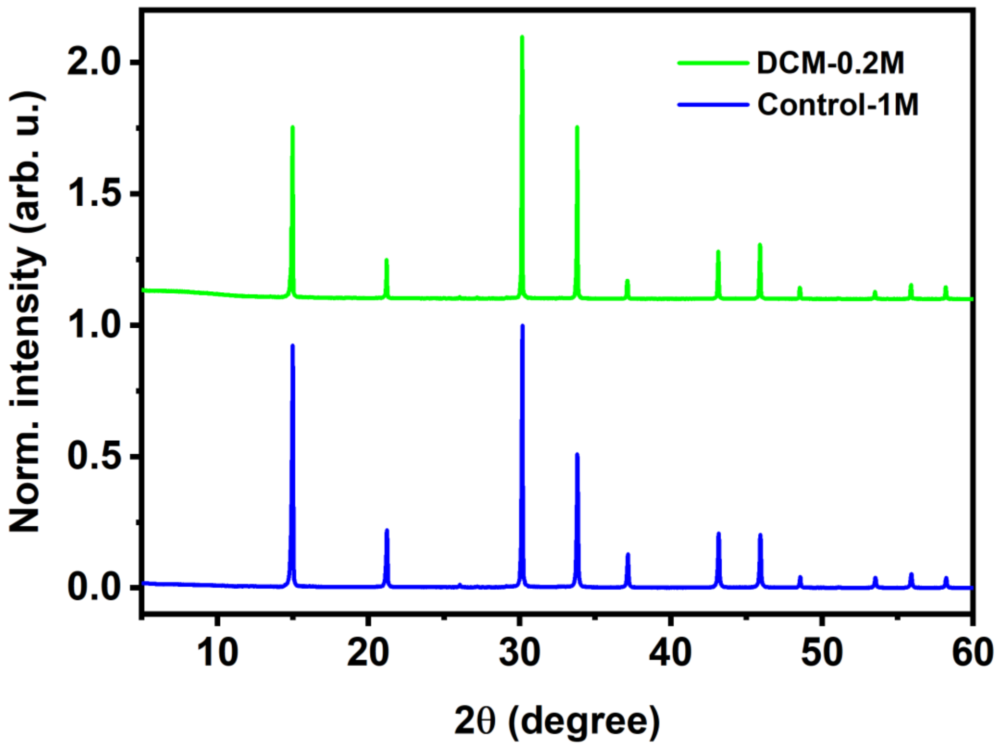

Figure S3. XRD patterns of ground Control-1M and DCM-0.2M $MAPbBr_3$ crystals.

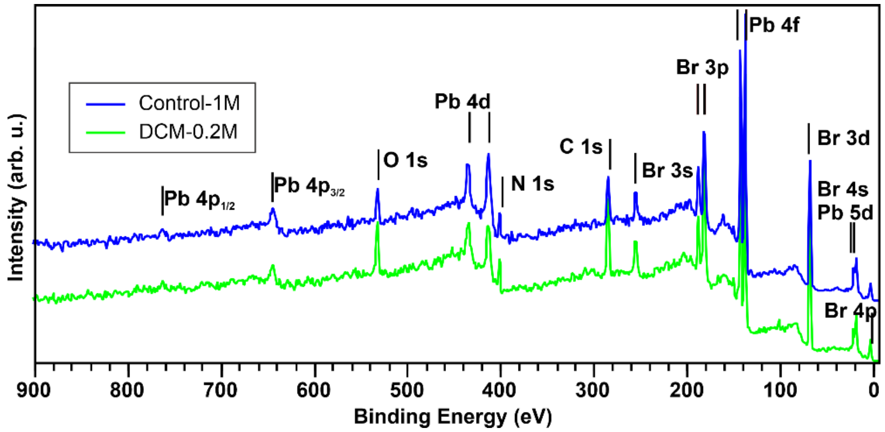

Figure S4. Broad XPS spectra of Control-1M and DCM-0.2M $MAPbBr_3$ crystals. There are well visible core level lines from the Pb, Br, C, N element which are included in the samples. There is one additional peaks O 1s from oxygen which adsorbed on the surfaces due to a sample transfer through air.

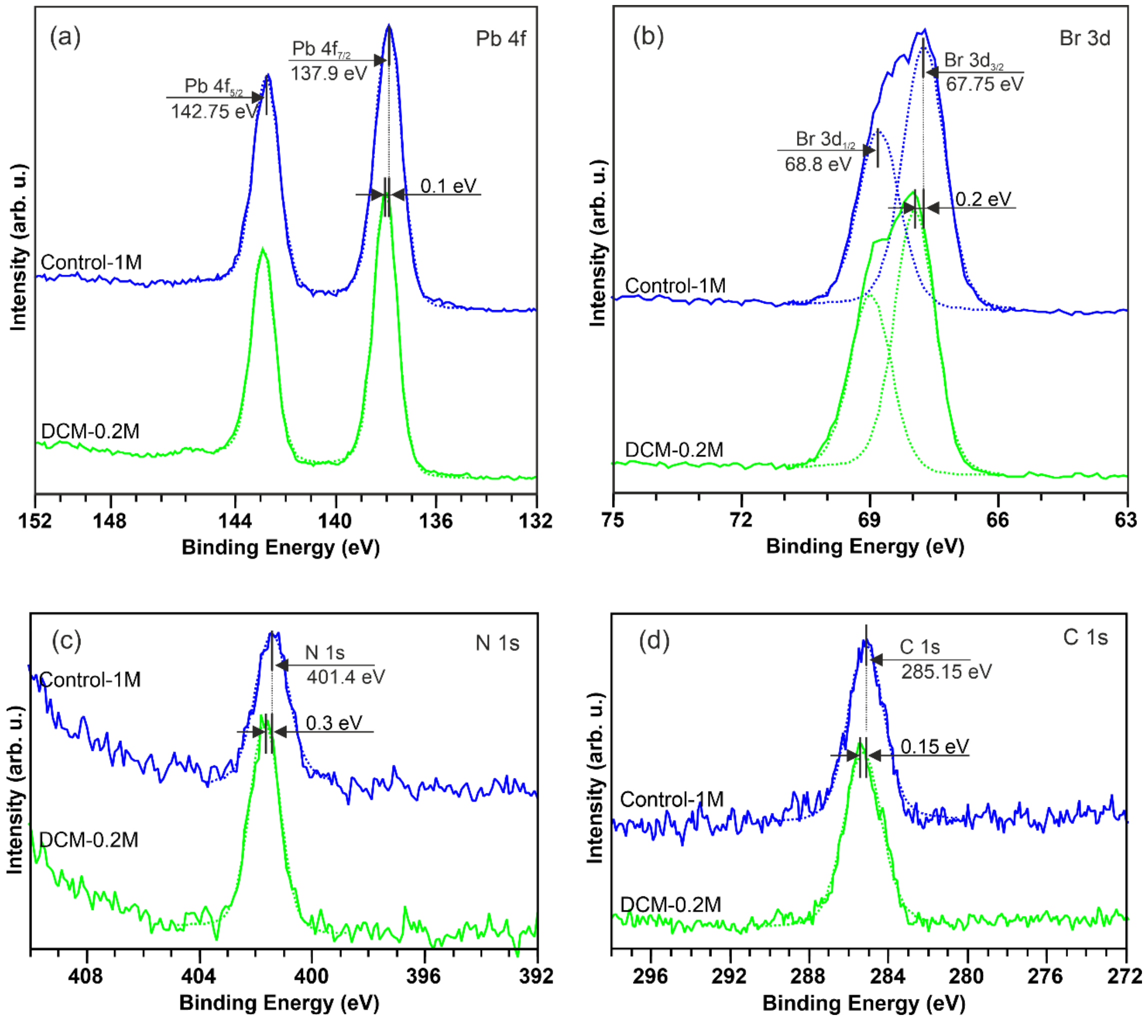

Figure S5. XPS spectra of (a) the Pb 4f, (b) Br 3d, (c) N 1s and (d) C1s core level lines for Control-1M and DCM-0.2M $MAPbBr_3$ crystals. Solid and dotted lines correspond to measured data and fitted peaks.

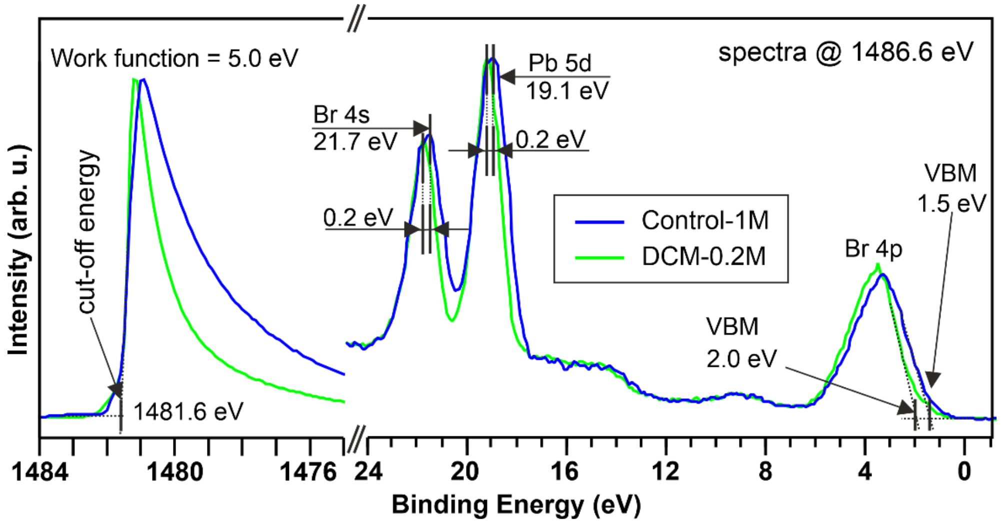

Figure S6. Valence band, cut-off energy and shallow core level line of Br 4s and Pb 5d for the for Control-1M and DCM-0.2M $MAPbBr_3$ crystals.

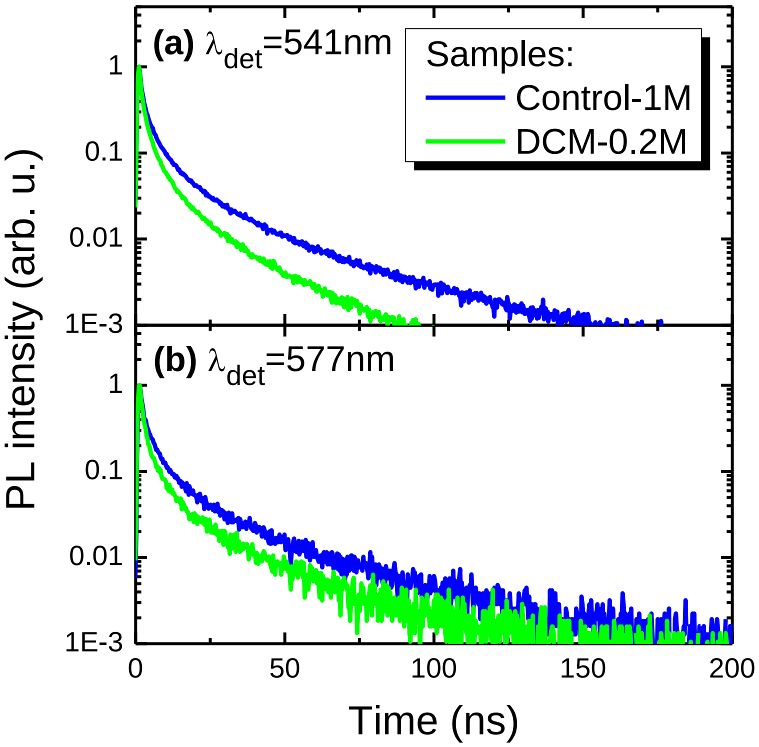

Figure S7. PL decay of defect-assisted emission at (a) 541 nm (b) 577 nm.

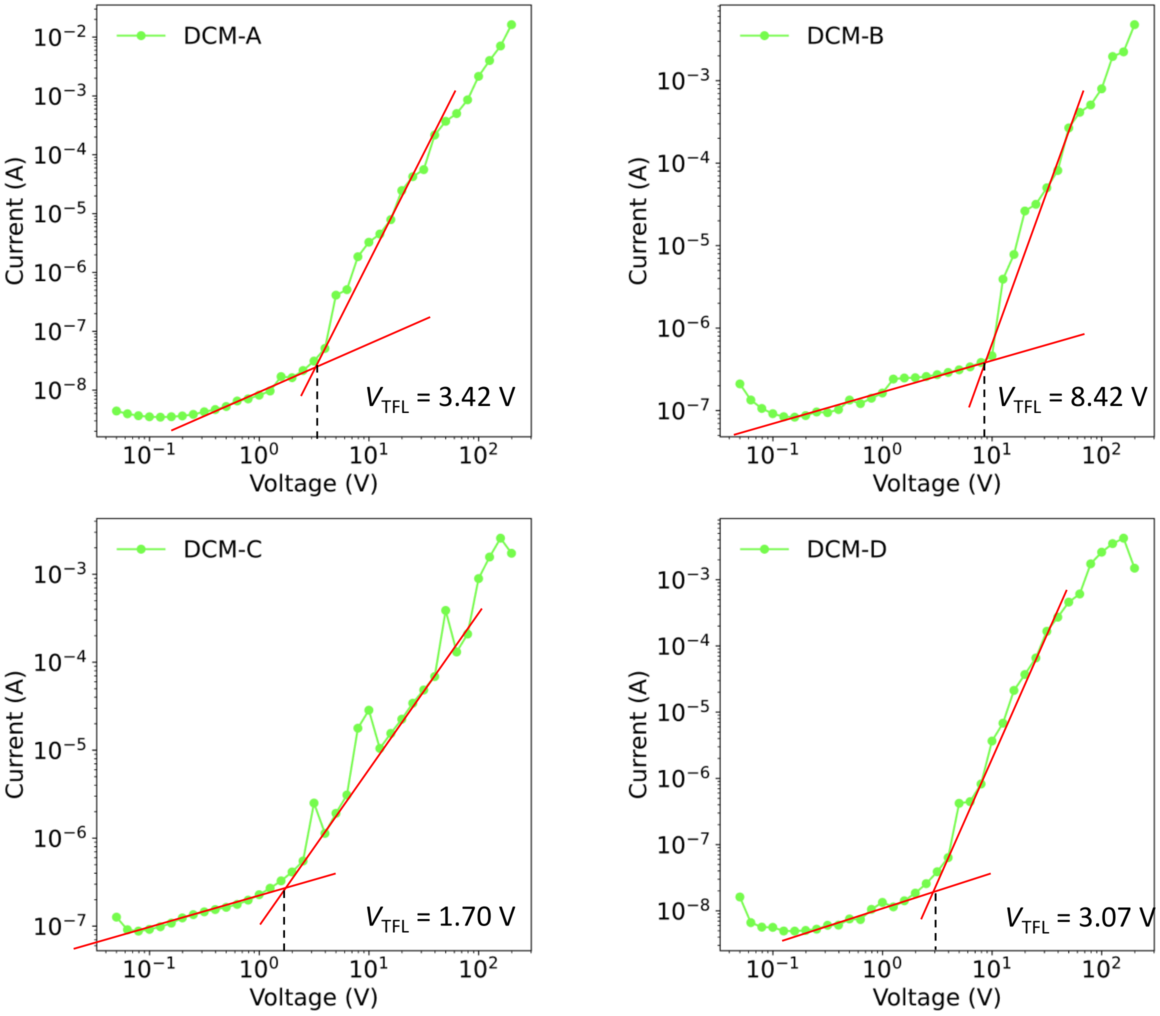

Figure S8. The log*I*-log*V* plots of DCM-0.2M crystals.

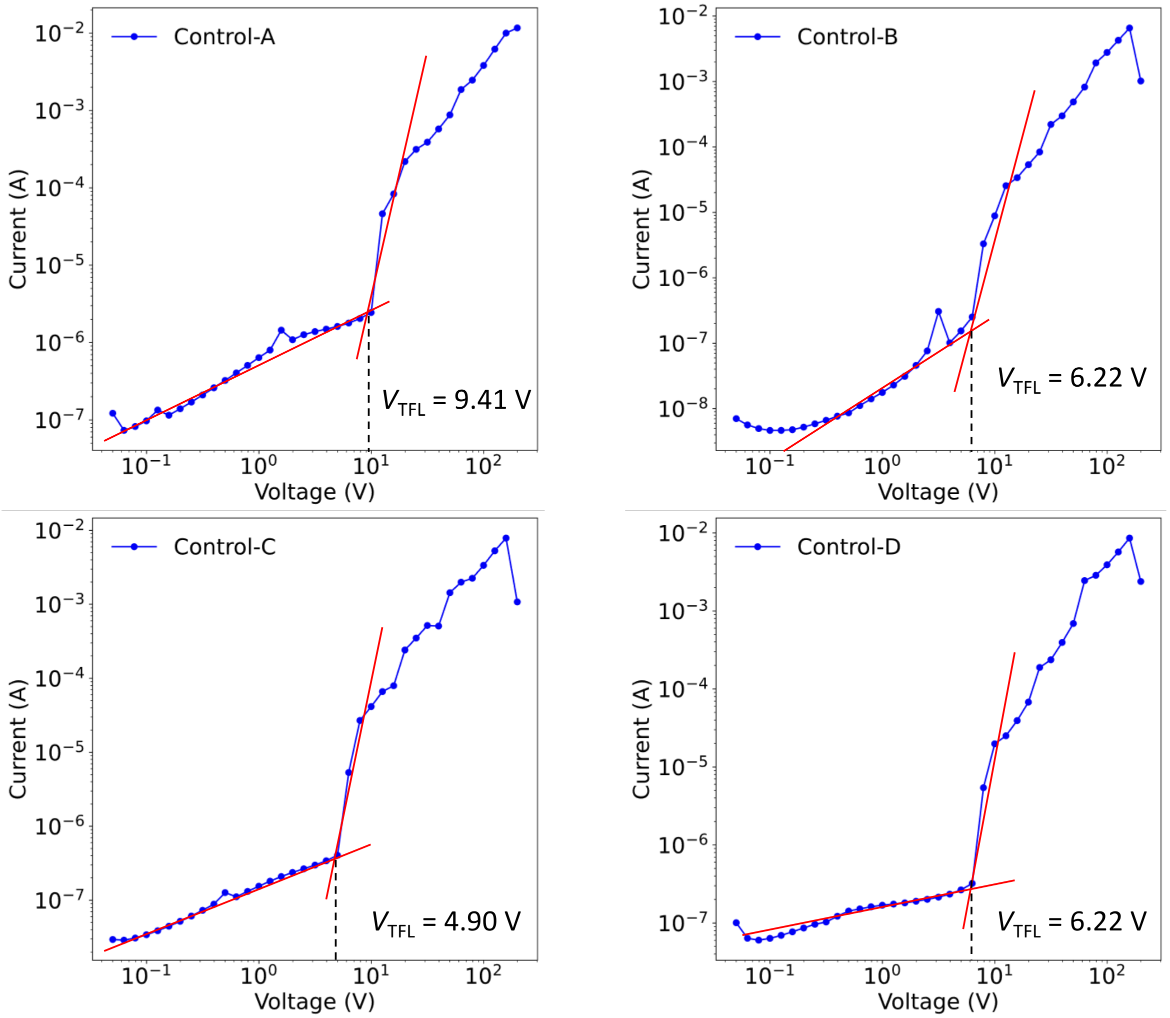

Figure S9. The log*I*-log*V* plots of Control-1M crystals.

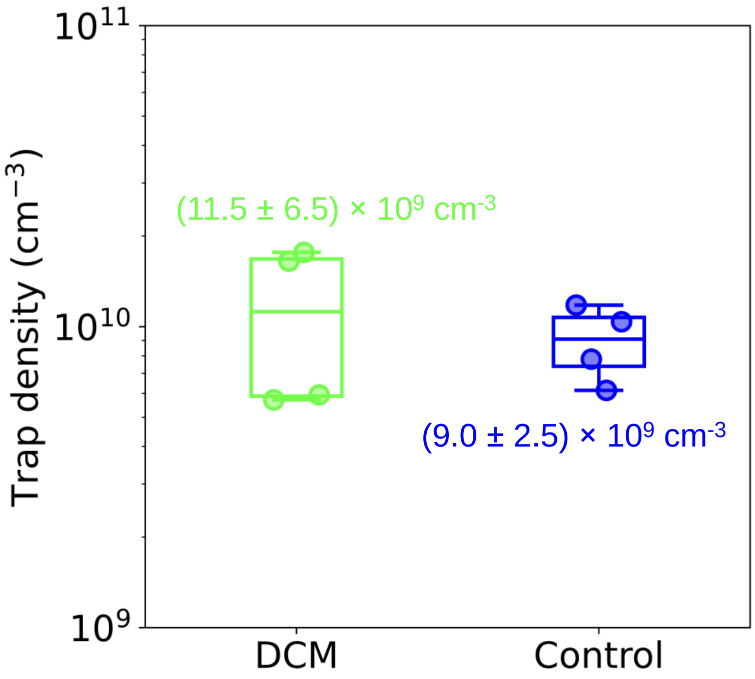

Figure S10. Trap density of DCM-0.2M and Control-1M crystals.

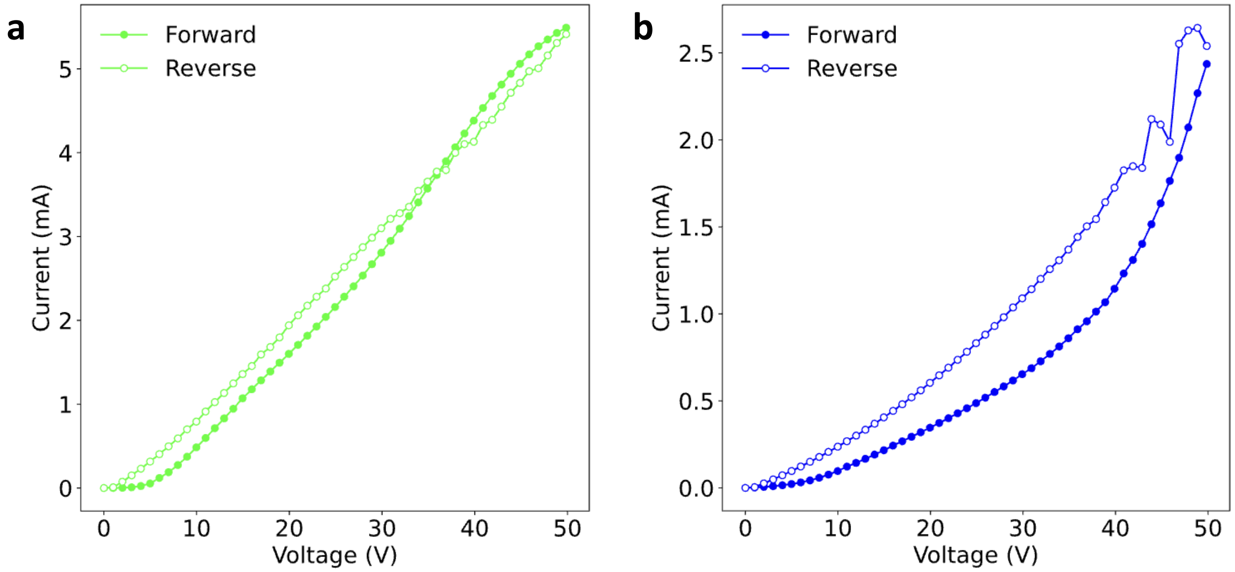

Figure S11. Hysteresis in the *I-V* curves of the devices. (a) DCM-0.2M (b) Control-1M. Continuous voltages were applied from 0 V to 50 V (forward scan) and from 50 V to 0 V (reverse scan) with a scan rate of 2 V/s. Both crystals have the same thickness of 1.3 mm.

To quantify the magnitude of the hysteresis, we defined a hysteresis factor as follows:

$$F_{hysteresis} = \frac{1}{n}\sum_{i=0}^{n}\left|\frac{I_F(V_{\mathrm{i}}) - I_R(V_{\mathrm{i}})}{I_F(V_{\mathrm{i}})}\right|$$

where n is the number of the data point, $I_F(V_{\mathrm{i}})$ and $I_R(V_{\mathrm{i}})$ are the normalized current at $V = V_{\mathrm{i}}$ during forward scan and reverse scan, respectively.

This equation gives $F_{\mathrm{hysteresis}}$ of 0.248 and 0.479 for DCM-0.2M and Control-1.0M crystals, respectively, indicating the DCM-0.2M has less hysteresis.

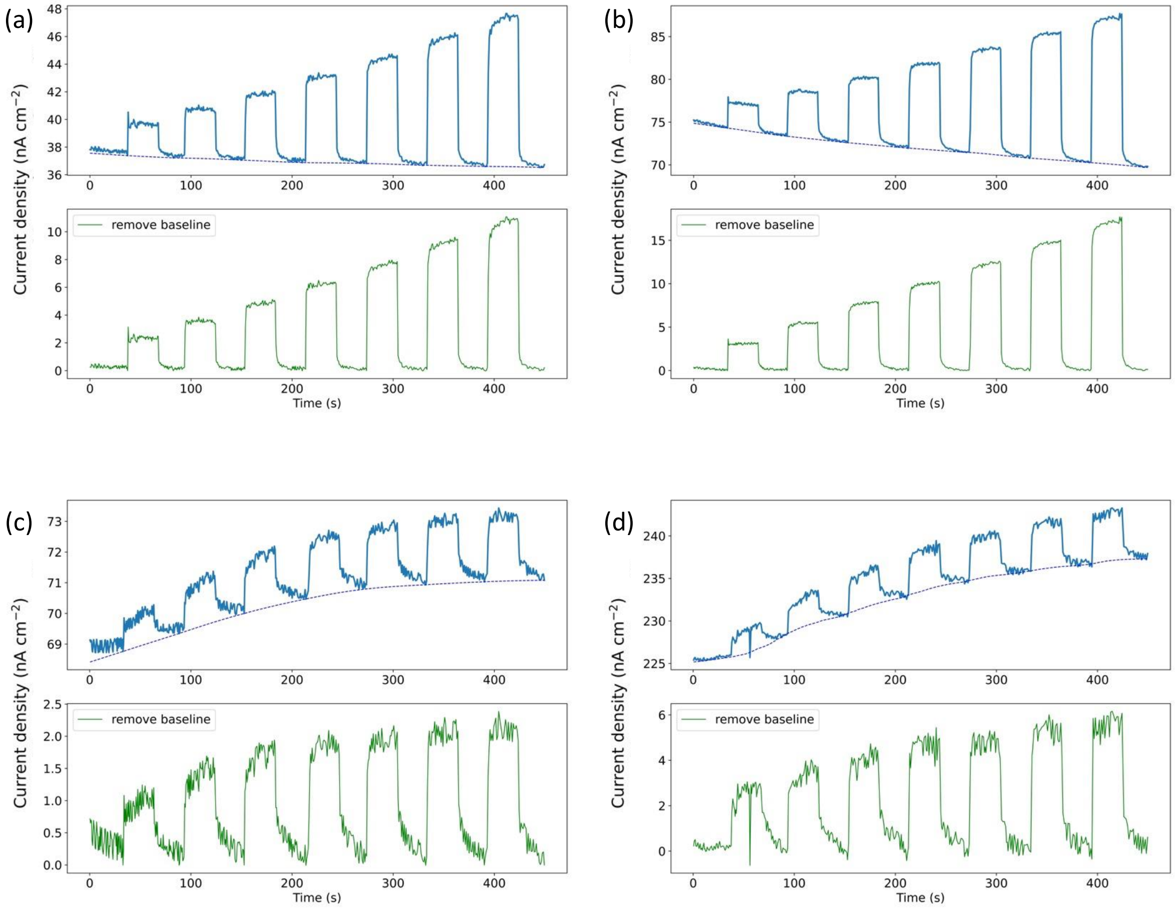

Figure S12. X-ray response currents (blue line), the baselines (dashed blue line), and the net response currents; response currents - baseline (green line). (a) DCM-0.2M at -2 V, (b) DCM-0.2M at -5 V, (c) Control-1M at -2 V, and (d) Control-1M at -5 V.

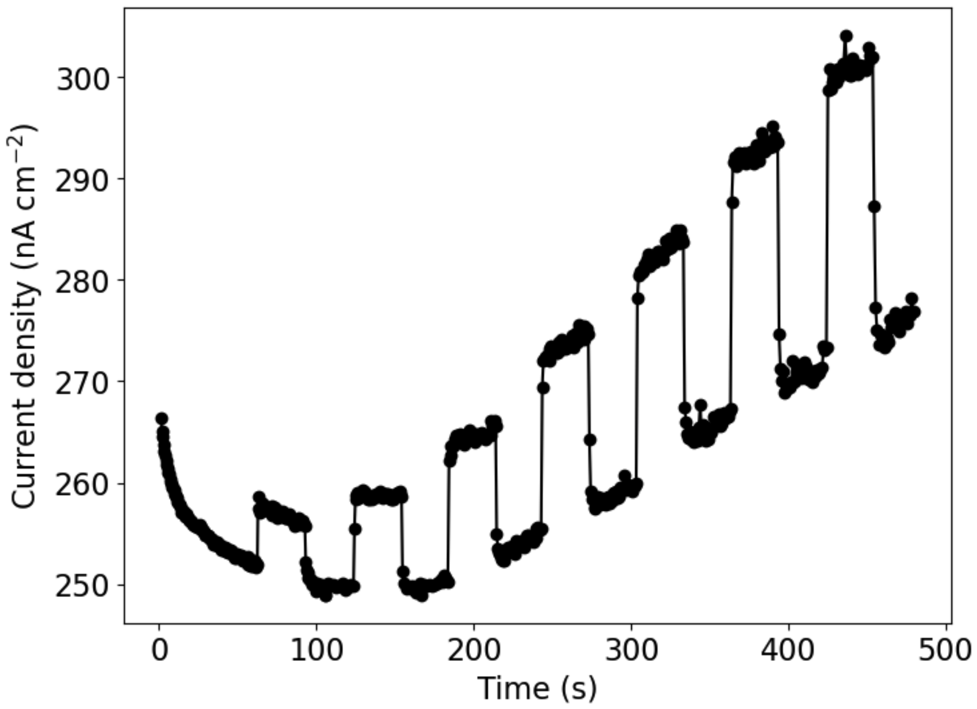

Figure S13. The response current of the DCM-0.2M device at the bias of -20V. The X-ray dose rates are the same as the ones in Figure 3c.

Table SI. Comparison of direct-conversion perovskite X-ray detectors.

Material	Thickness (mm)	Device structure	kVp	Electric field (V mm^{-1})	Sensitivity (μC Gy_{air}^{-1} cm^{-2})	Ref
$MAPbBr_3$ single crystal	2	Au/PVK/C_{60}/BCP/Ag or Au	-	-	80	1
$MAPbI_3$ film	0.83	ITO/PI PVK/PVK/PI PVK/TFT/ITO	100	240	11,000	2
$MAPbBr_{2.94}Cl_{0.06}$ single crystal	1	Cr/C_{60}/BCP/PVK/Cr	8	60	84,000	3
$MAPbBr_3$ single crystal	2	Si/PVK/C_{60}/BCP/Au	50	0.5	322	4
$MAPbI_3$ wafer	1	Ag/ZnO/PCBM/PVK/PEDOT: PSS/ITO	70	200	2527	5
$MAPbBr_3$ single crystal	7	Au/poly-TPD/PVK/C_{60}/PCBM/Ag	30	14.3	23,600	6
$CsPbBr_3$ film	0.24	FTO/PVK/Au	50	5	55,684	7
$CsPbBr_3$ film	0.018	Au/PVK/Pt	35	110	1,700	8
$MAPbBr_3$ single crystal	2	AZO/PVK/Au	80	5	529	9
$MAPbBr_3$ single crystal	2.6	Al/PVK/Au	50	14300	359	10
$MAPbBr_3$ single crystal	3	Au/PVK/Au	39	0.83	259.9	11
$MAPbBr_3$ single crystal	-	Cr/BCP/C60/PVK/Au	120	-	3,928.3	12
$MAPbI_3$ wafer	0.8	Au/PCBM/PVK/Au	40	12.5	122,000	13
$MAPbBr_3$ single crystal	2.02	InGa/C_{70}/PVK/Au	-	1.98	184.6	14
$MAPb(I_{0.9}Cl_{0.1})_3$-filled membrane	0.24	Cr/BCP/C_{60}/PVK/Cr	50	50	2,204	15
$Cs_2AgBiBr_6$ film	0.092	W/PVK/Pt	70	109	487	16
$MAPbBr_3$ film/$MAPbBr_3$ single crystal	2	Au/PVK/Ga	40	5	549.9	This work

References

(1) Wei, H.; Fang, Y.; Mulligan, P.; Chuirazzi, W.; Fang, H.-H.; Wang, C.; Ecker, B. R.; Gao, Y.; Loi, M. A.; Cao, L.; Huang, J. Sensitive X-Ray Detectors Made of Methylammonium Lead Tribromide Perovskite Single Crystals. *Nat. Photon* **2016,** *10*, 333-339.

(2) Kim, Y. C.; Kim, K. H.; Son, D.-Y.; Jeong, D.-N.; Seo, J.-Y.; Choi, Y. S.; Han, I. T.; Lee, S. Y.; Park, N.-G. Printable Organometallic Perovskite Enables Large-Area, Low-Dose X-Ray Imaging. *Nature* **2017,** *550*, 87.

(3) Wei, H.; DeSantis, D.; Wei, W.; Deng, Y.; Guo, D.; Savenije, T. J.; Cao, L.; Huang, J. Dopant Compensation in Alloyed $CH_3NH_3PbBr_{3-X}Cl_x$ Perovskite Single Crystals for Gamma-Ray Spectroscopy. *Nat. Mater.* **2017,** *16*, 826-833.
(4) Wei, W.; Zhang, Y.; Xu, Q.; Wei, H.; Fang, Y.; Wang, Q.; Deng, Y.; Li, T.; Gruverman, A.; Cao, L.; Huang, J. Monolithic Integration of Hybrid Perovskite Single Crystals with Heterogenous Substrate for Highly Sensitive X-Ray Imaging. *Nat. Photonics* **2017,** *11*, 315-321.
(5) Shrestha, S.; Fischer, R.; Matt, G. J.; Feldner, P.; Michel, T.; Osvet, A.; Levchuk, I.; Merle, B.; Golkar, S.; Chen, H.; Tedde, S. F.; Schmidt, O.; Hock, R.; Rührig, M.; Göken, M.; Heiss, W.; Anton, G.; Brabec, C. J. High-Performance Direct Conversion X-Ray Detectors Based on Sintered Hybrid Lead Triiodide Perovskite Wafers. *Nat. Photon* **2017,** *11*, 436-440.
(6) Wang, X.; Zhao, D.; Qiu, Y.; Huang, Y.; Wu, Y.; Li, G.; Huang, Q.; Khan, Q.; Nathan, A.; Lei, W.; Chen, J. Pin Diodes Array Made of Perovskite Single Crystal for X-Ray Imaging. *physica status solidi (RRL)* **2018,** *12*, 1800380.
(7) Pan, W.; Yang, B.; Niu, G.; Xue, K.-H.; Du, X.; Yin, L.; Zhang, M.; Wu, H.; Miao, X.-S.; Tang, J. Hot-Pressed $CsPbBr_3$ Quasi-Monocrystalline Film for Sensitive Direct X-Ray Detection. *Adv. Mater.* **2019,** *31*, 1904405.
(8) Gou, Z.; Huanglong, S.; Ke, W.; Sun, H.; Tian, H.; Gao, X.; Zhu, X.; Yang, D.; Wangyang, P. Self-Powered X-Ray Detector Based on All-Inorganic Perovskite Thick Film with High Sensitivity under Low Dose Rate. *physica status solidi (RRL)* **2019,** *13*, 1900094.
(9) Li, L.; Liu, X.; Zhang, H.; Zhang, B.; Jie, W.; Sellin, P. J.; Hu, C.; Zeng, G.; Xu, Y. Enhanced X-Ray Sensitivity of $MAPbBr_3$ Detector by Tailoring the Interface-States Density. *ACS Appl. Mater. Interfaces* **2019,** *11*, 7522-7528.
(10) Xu, Q.; Shao, W.; Li, Y.; Zhang, X.; Ouyang, X.; Liu, J.; Liu, B.; Wu, Z.; Ouyang, X.; Tang, X.; Jia, W. High-Performance Surface Barrier X-Ray Detector Based on Methylammonium Lead Tribromide Single Crystals. *ACS Appl. Mater. Interfaces* **2019,** *11*, 9679-9684.
(11) Geng, X.; Feng, Q.; Zhao, R.; Hirtz, T.; Dun, G.; Yan, Z.; Ren, J.; Zhang, H.; Liang, R.; Tian, H.; Xie, D.; Yang, Y.; Ren, T.-L. High-Quality Single Crystal Perovskite for Highly Sensitive X-Ray Detector. *IEEE Electron Device Lett.* **2020,** *41*, 256-259.
(12) Song, J.; Feng, X.; Li, H.; Li, W.; Lu, T.; Guo, C.; Zhang, H.; Wei, H.; Yang, B. Facile Strategy for Facet Competition Management to Improve the Performance of Perovskite Single-Crystal X-Ray Detectors. *J. Phys. Chem. Lett.* **2020,** *11*, 3529-3535.

(13) Hu, M.; Jia, S.; Liu, Y.; Cui, J.; Zhang, Y.; Su, H.; Cao, S.; Mo, L.; Chu, D.; Zhao, G.; Zhao, K.; Yang, Z.; Liu, S. F. Large and Dense Organic-Inorganic Hybrid Perovskite $CH_3NH_3PbI_3$ Wafer Fabricated by One-Step Reactive Direct Wafer Production with High X-Ray Sensitivity. *ACS Appl. Mater. Interfaces* **2020,** *12*, 16592-16600.
(14) Yao, F.; Peng, J.; Li, R.; Li, W.; Gui, P.; Li, B.; Liu, C.; Tao, C.; Lin, Q.; Fang, G. Room-Temperature Liquid Diffused Separation Induced Crystallization for High-Quality Perovskite Single Crystals. *Nat. Commun.* **2020,** *11*, 1194.
(15) Zhao, J.; Zhao, L.; Deng, Y.; Xiao, X.; Ni, Z.; Xu, S.; Huang, J. Perovskite-Filled Membranes for Flexible and Large-Area Direct-Conversion X-Ray Detector Arrays. *Nat. Photonics* **2020,** *14*, 612-617.
(16) Haruta, Y.; Wada, S.; Ikenoue, T.; Miyake, M.; Hirato, T. Columnar Grain Growth of Lead-Free Double Perovskite Using Mist Deposition Method for Sensitive X-Ray Detectors. *Cryst. Growth Des.* **2021,** *21*, 4030-4037.
