# Supplementary material for: Surface Engineering of Methylammonium Lead Bromide Perovskite Crystals for Enhanced X-ray Detection
Source: J Phys Chem Lett. 2023 Oct 5;14(40):9136–44. doi: 10.1021/acs.jpclett.3c02061 (PMC10577767; doi:10.1021/acs.jpclett.3c02061)
Supplement: Supplementary file 2 — jz3c02061_si_002.pdf [file jz3c02061_si_002.pdf]

jz-2023-02061y.R1

Name: Peer Review Information for "Surface Engineering of Methylammonium Lead Bromide Perovskite Crystals for Enhanced X-ray Detection"

First Round of Reviewer Comments

Reviewer: 1

Comments to the Author

The authors represent a significant new contribution in the research area of X-ray detection using $MAPbBr_3$ and should be published as is. The XRD, TRPL, XPS, and I-V characterization show clear evidence of superior X-ray detection properties together with low density of the surface defects. Therefore, I strongly recommend this manuscript to be published without further revision.

Reviewer: 2

Comments to the Author

In this paper, the authors demonstrated improved device performance of $MAPbBr_3$ single crystal based X-ray detector using surface engineering method. It is clear that there is an effect on the surface of $MAPbBr_3$ single crystal by slow diffusion of DCM anti-solvent. However, bulk properties are more important than surface properties for X-ray detectors,. Moreover, an in-depth understanding of the improved performance is missing. Major revisions in the presentation, argumentation, experiments, and literature citations are required. The most important issues are:

1. The authors stated that the polycrystalline $MAPbBr_3$ film is formed on the crystal surface. Is it really compact and pinhole-free film? What is the thickness of this film? Also, by varying the thickness can we tune the detection properties?

2. The authors stated that DCM-0.2M showed bright green emission under UV light, while Control-1M crystal was found to be non-emissive under UV light. This was attributed to the as-formed polycrystalline layer on the surface. However, the authors should show that the polycrystalline $MAPbBr_3$ thin film indeed can be emissive under the same UV light.

3. It was stated that “Both the top and bottom surfaces of the DCM-0.2M crystals appear to be rougher with respect to the Control-1M crystals” Could the authors experimentally confirm the different roughness of these crystals e.g. by AFM?

4. What about trap density? The authors should experimentally measure, calculate and compare the trap density of both crystals by SCLC method. For reproducibility, measuring at least 3-5 different individual crystals is recommended.

5. A comparison Table of photodetection properties of X-rays detectors should be made for the reported in this draft and reported by others lead halide perovskite SCs based photodetectors.

6. Passivation of SC surface may have a significant effect on ion migration. Is it possible to study the impact of the proposed passivation on the activation energy of MAPbBr3 SC?

7. Figure 3b shows only a dark I-V plot of both devices. Please provide the relevant I-V plot of both devices under 25.2 μGyair s-1.

8. There is no information related to Au electrode thickness. For X-ray measurement, the thickness of the top electrode is essential.

9. In principle, X-ray detectors are working under applied high bias. Why the authors did not test the device under high ±40 V?

10. Some relevant literature works on the passivation of MAPbBr3 SC (Journal of Applied Physics 127, 185501 (2020)) and MAPbBr3 SC based photodetectors (ACS Photonics 2023, 10, 1424) are missing.

Reviewer: 3

Comments to the Author

The authors provided alternative synthetic strategy to improve the MAPbBr3 single crystal with effective surface passivation to demonstrate overall quality of the single crystal, by presenting the intense green UV light, elongated PL decay lifetime, lower defect density with 5 times higher X-ray detecting ablity. Through XPS, TRPL and dark J-V curves, the authors presented their crystals have better crystalline quality by decreasing the surface defect density. However, considering the manuscript scope, the description towards to demonstrate higher crystalline quality for better X-ray detecting ability, it seems like the manuscript would be re-considered where to be published in the Journal of Physical Chemistry Letters due to mismatch journal aim and the flow/purpose of this manuscript. Overall supporting characterizations could support the enhanced quality of the crystal, so this manuscript could be considered to more suitable aim, such as Crystal Growth & Design, or any of crystal focused journals.

Also, the authors could be consider the following aspects for the manuscript, as follows:

1. In page 8, line 56, there is no Figure2e in the manuscript. The authors should check again about the description and/or typo.

2. Like as the experiment shown in Figure 3a and 3b, the authors may provide defect density differences through Space Charge Limited Current (SCLC) method. To demonstrate the enhanced crystalline quality as well as decreasing the defect density in both surface and inside of the crystal, more detailed analysis would need to be proposed.

Author's Response to Peer Review Comments:

Dear Prof.

Thank you for your email on August 10, 2023 regarding our manuscript "***Surface Engineering of Methylammonium Lead Bromide Perovskite Crystals for Enhanced X-ray Detection".*** We are grateful to all of the reviewers for time spent reviewing our manuscript. You will see in the next paragraphs that we have fully addressed all of the suggestions and comments made by the reviewers, improving our presentation throughout the manuscript.

We are grateful to the *Journal of Physical Chemistry Letters* Editorial Office for their handling of our manuscript. We are confident that we have fully addressed each point raised by the referees appropriately.

Yours Sincerely,

Ahmed L. Abdelhady
Chemistry department – Khalifa University – UAE

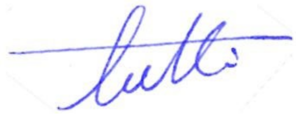

Reviewer: 1

Recommendation: This paper represents a significant new contribution and should be published as is.

Comments:

The authors represent a significant new contribution in the research area of X-ray detection using MAPbBr3 and should be published as is. The XRD, TRPL, XPS, and I-V characterization show clear evidence of superior X-ray detection properties together with low density of the surface defects. Therefore, I strongly recommend this manuscript to be published without further revision.

Our Response: We thank the reviewer for their comments.

Reviewer: 2

Recommendation: This paper may be publishable, but major revision is needed; I would like to be invited to review any future revision.

Comments:

In this paper, the authors demonstrated improved device performance of MAPbBr3 single crystal based X-ray detector using surface engineering method. It is clear that there is an effect on the surface of MAPbBr3 single crystal by slow diffusion of DCM anti-solvent. However, bulk properties are more important than surface properties for X-ray detectors,. Moreover, an in-depth understanding of the improved performance is missing. Major revisions in the presentation, argumentation, experiments, and literature citations are required. The most important issues are:

1. The authors stated that the polycrystalline MAPbBr3 film is formed on the crystal surface. Is it really compact and pinhole-free film? What is the thickness of this film? Also, by varying the thickness can we tune the detection properties?

Our Response: We thank the reviewer for this insightful comment. We reckon that the film is compact however may contain pinholes (see Figure R1 below). It is hard to confirm if the core crystal of the crystal is being exposed or not. Furthermore, as the thin layer on top of the crystal is formed in-situ, the control of the layer thickness is relatively challenging. Nevertheless, we are working on optimizing the crystallization technique for controlled top layer thickness along with utilizing FIB-SEM for precise measurement of the film thickness. We believe all this will be part of a follow up study.

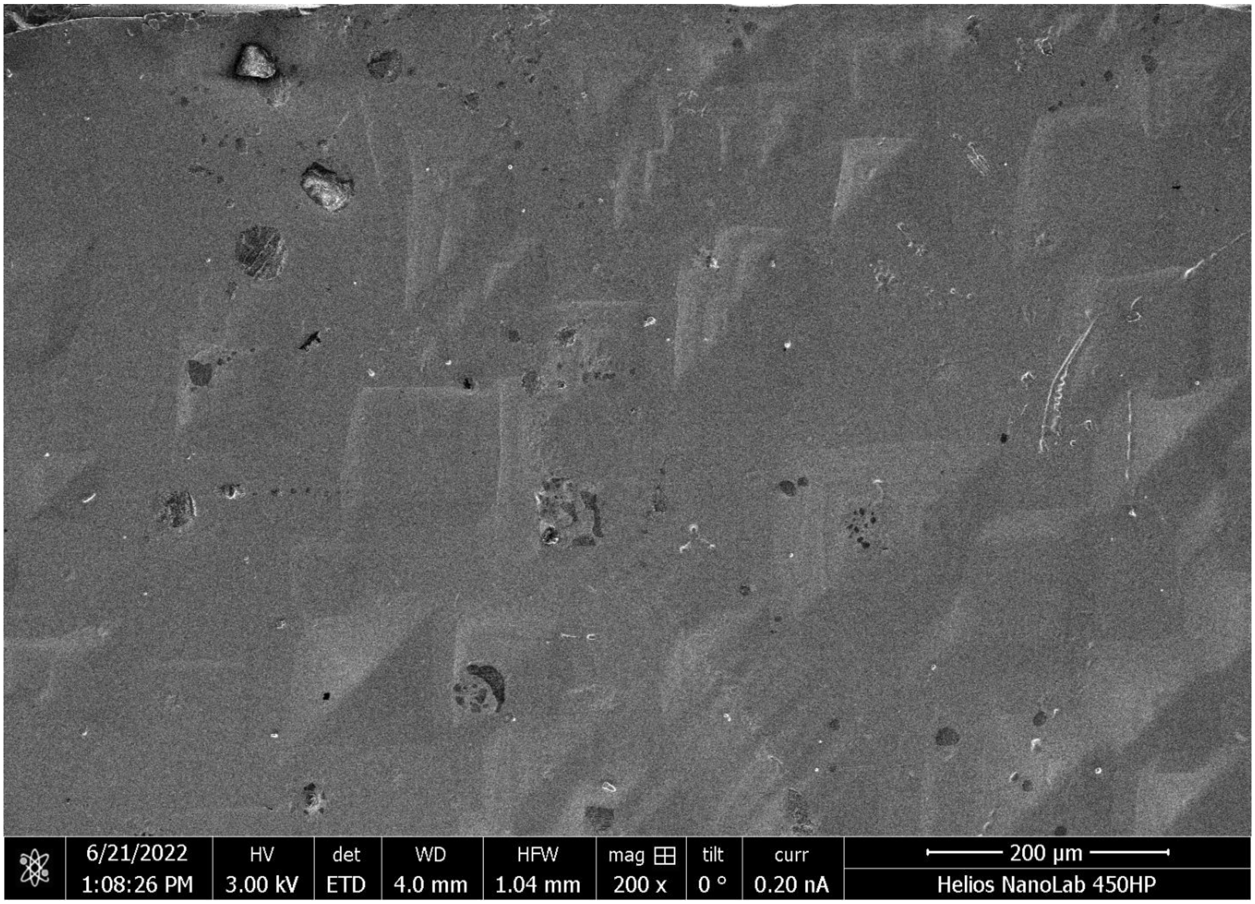

Figure R1. Scanning electron microscopy image of the DCM-0.2M crystal showing that the top surface layer might not be compact or pinhole-free.

2. The authors stated that DCM-0.2M showed bright green emission under UV light, while Control1M crystal was found to be non-emissive under UV light. This was attributed to the as-formed polycrystalline layer on the surface. However, the authors should show that the polycrystalline MAPbBr3 thin film indeed can be emissive under the same UV light.

Our Response: We have attempted the fabrication of $MAPbBr_3$ thin films using our crystallization technique. Under UV light the polycrystalline thin film showed green emission as demonstrated in Figure R2. Furthermore, it has been previously reported in literature that $MAPbBr_3$ or $FAPbBr_3$ polycrystalline thin films are green emissive under UV light. See Lee et al *ACS Nano* 2017, 11, 3311–3319 and Xing et al *J. Phys. Chem. Lett.* 2022, 13, 704–710.

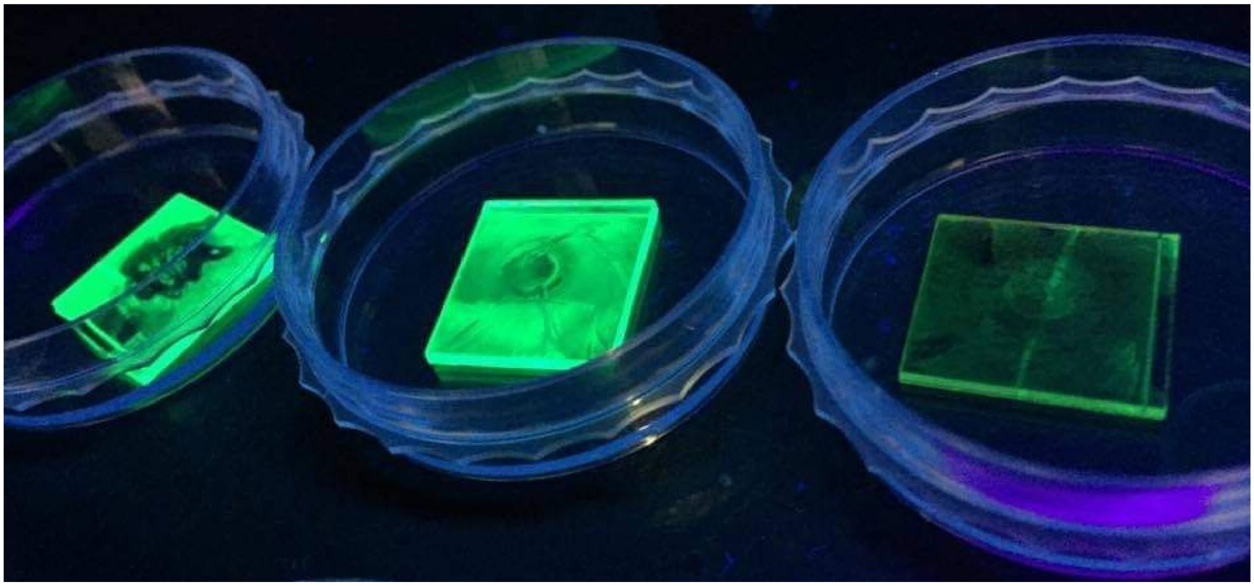

Figure R2. Photographs of $MAPbBr_3$ thin films under UV light displaying green emission. Films were fabricated by spin coating the solvent acidolysis crystallization precursor solution.

3. It was stated that “Both the top and bottom surfaces of the DCM-0.2M crystals appear to be rougher with respect to the Control-1M crystals” Could the authors experimentally confirm the different roughness of these crystals e.g. by AFM?

Our Response: Following the reviewers comment, we have performed AFM measurement. In line with the optical microscopy images and SEM images, AFM measurements confirmed that the surface of the DCM-0.2M crystals are rougher than the Control-1M crystals.

We have included the text and figure below in the revised version. See Page 4 in the revised manuscript and Figure S1.

Figure S1a-c in the supporting information represents atomic force microscopy (AFM) results of the Control-1M $MAPbBr_3$ SC bottom face, while Figure S1d-f shows the corresponding images for the DCM-0.2M SC. The AFM analysis was performed in different places on the sample surface as well as with different scan sizes. Comparing a 5×5 μm^2 scan for both samples (Figure S1a and S1d), the higher surface root-mean-square (RMS) roughness was observed in case of DCM-0.2M SC. Bottom face of the Control-1M $MAPbBr_3$ SC exhibits step-like areas with RMS equal to 2.1 nm. In case of the DCM-0.2M SC, terrace-like surface is also observed, however with RMS equal to 7.6 nm. Figure S1b and S1e show smaller size AFM images measured in other regions than the one presented in Figure S1a and S1d, for Control-1M and DCM-0.2M SCs, respectively. For these images the line scan profile was extracted, which allowed for detailed roughness analysis. In Figure S1c, representing the line scan profile for the Control-1M $MAPbBr_3$ SC, steps with height up to ~

10 nm can be observed. Line scan profile for DCM-0.2M SC, presented in Figure S1f, shows terraces with height up to ~40 nm. These AFM results concluded that the DCM-0.2M SCs exhibits higher surface roughness with respect to the Control-1M SCs.

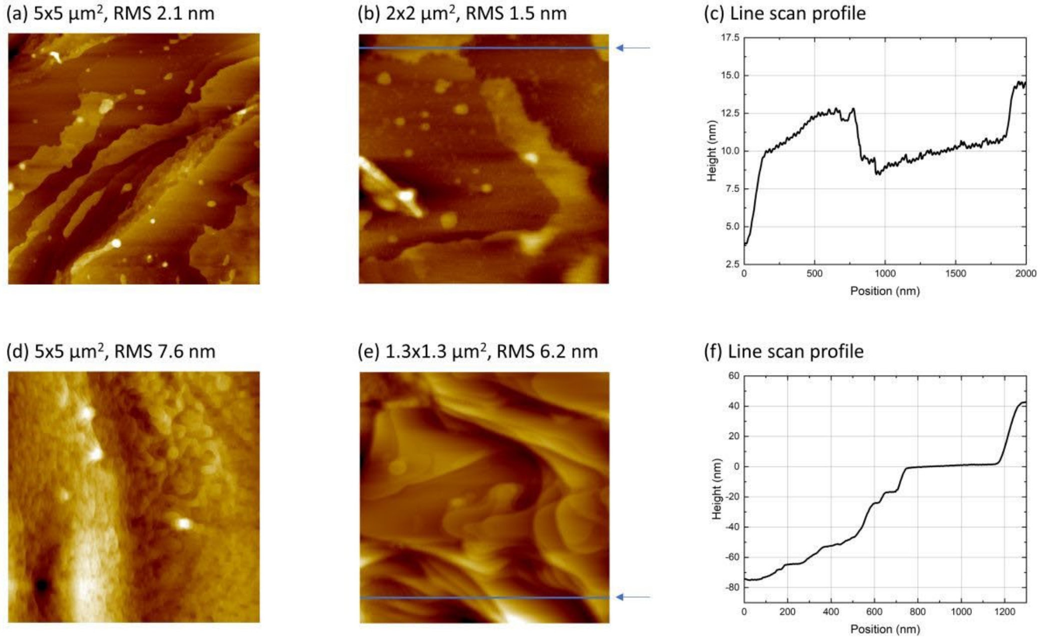

Figure S1. (a–c) AFM images with corresponding scan areas, roughness parameters and line scan profile of the Control-1M $MAPbBr_3$ SC bottom face. (d–f) Corresponding AFM images of the DCM-0.2M SC bottom face.

4. What about trap density? The authors should experimentally measure, calculate and compare the trap density of both crystals by SCLC method. For reproducibility, measuring at least 3-5 different individual crystals is recommended.

Our Response: We measured the trap density of both crystals using four crystals of each. Both crystals showed similar trap densities. We think this is because SCLC gives bulk property rather than surface property. However, PL results still suggest the DCM-0.2M crystals have fewer defects at the surface. Furthermore, we observed less hysteresis in the DCM-0.2M device compared to the Control-1.0M, suggesting less charge accumulation at the interface between perovskite and electrodes, which means a better surface of the DCM-0.2M crystal. These results suggest that DCM-0.2M and Control-1M have similar trap densities in the bulk however; DCM0.2M has less defect at the surface. Thus, we may conclude that the surface properties significantly affect the performance of the X-ray detectors using perovskite single crystals even if the bulk properties are similar, in agreement with the previous research reporting the improvement in the device performance by post-surface treatment (Chen et al. *ACS Appl. Mater. Interfaces* **2022,** *14*, 10917-10926).

We have included the text and figure below in the revised version. See Page 10 in the revised manuscript and Figure S8-11.

The trap densities of both crystals were measured by using 4 crystals of each with the pulsedvoltage space-charge limited current (PV-SCLC) method (Figures S8 and S9).[61] As shown in Figure S10, both crystals showed similar trap densities (DCM-0.2M: 11.5×10^9 cm^{-3}, Control-1M: 9.0×10^9 cm^{-3} on average). The trap density obtained by PV-SCLC reflects the bulk property rather than the surface property. On the other hand, when we applied continuous voltages to the devices, we observed less hysteresis in the DCM-0.2M device compared to the Control-1.0M (Figure S11), suggesting less charge accumulation at the interface between perovskite and electrodes, which indicates a better surface of the DCM-0.2M crystal. These results suggest that DCM-0.2M and Control-1M have similar trap densities in the bulk however; DCM-0.2M has less defect at the surface.

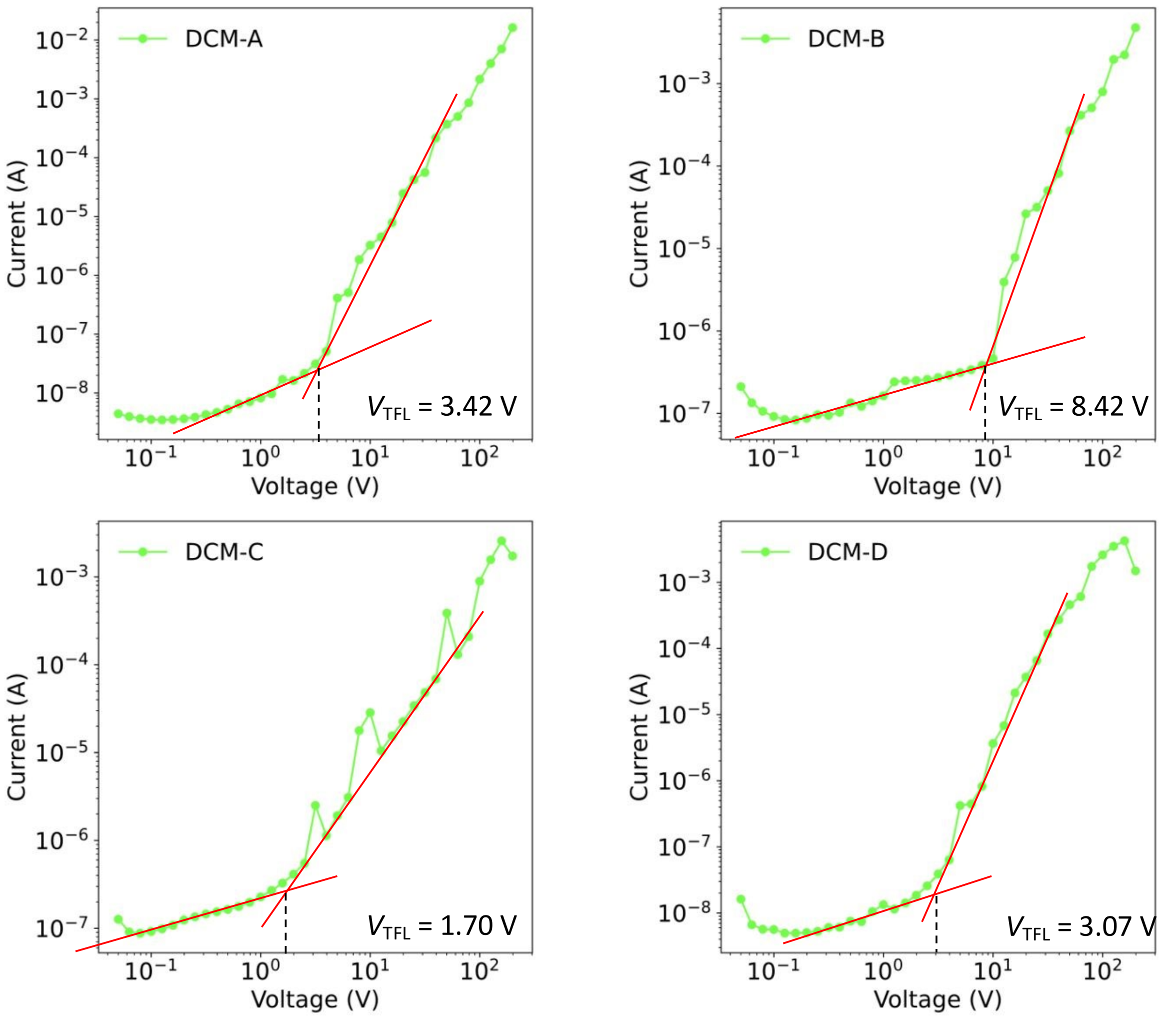

Figure S8. The log*I*-log*V* plots of DCM-0.2M crystals.

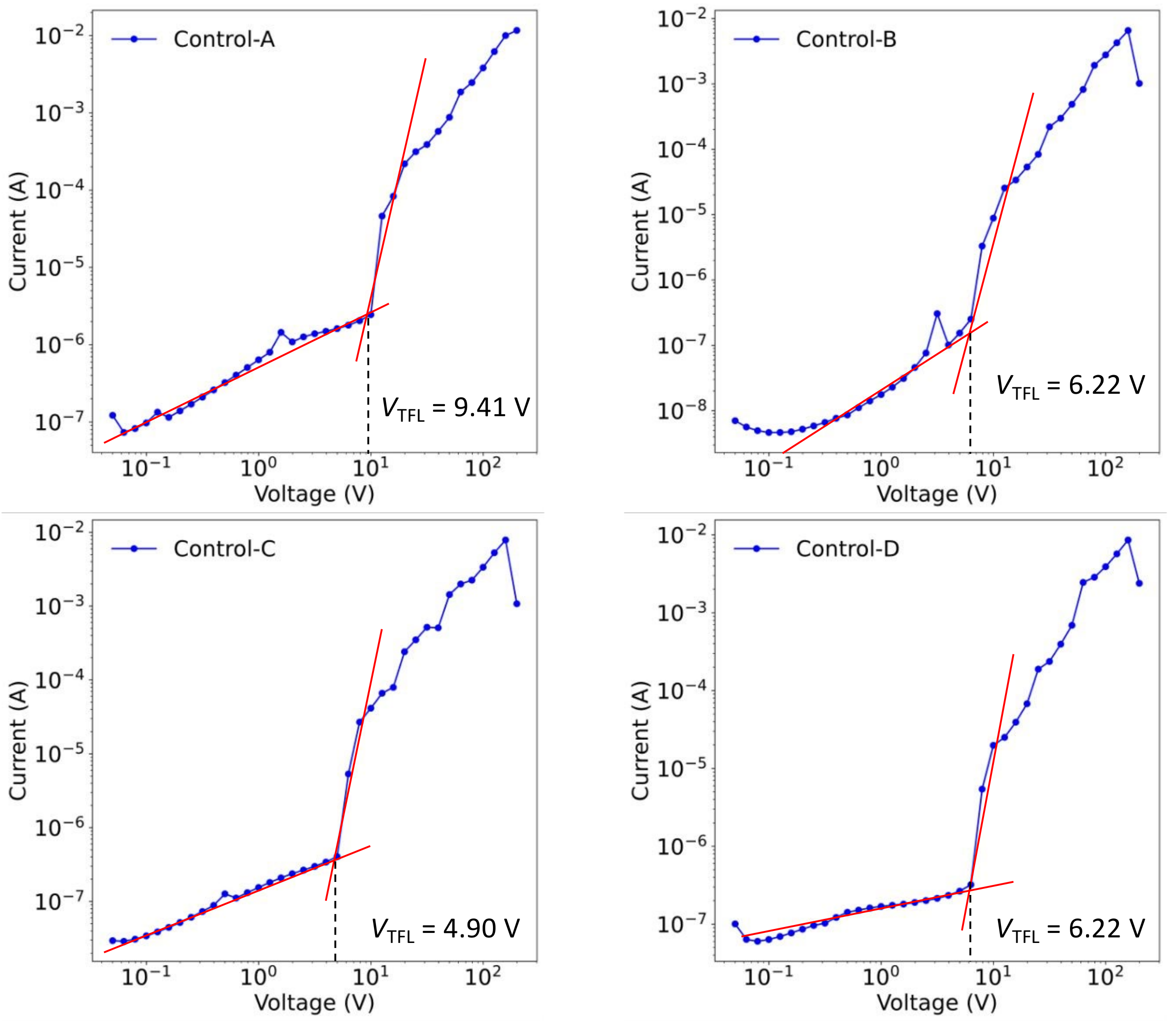

Figure S9. The log*I*-log*V* plots of Control-1M crystals.

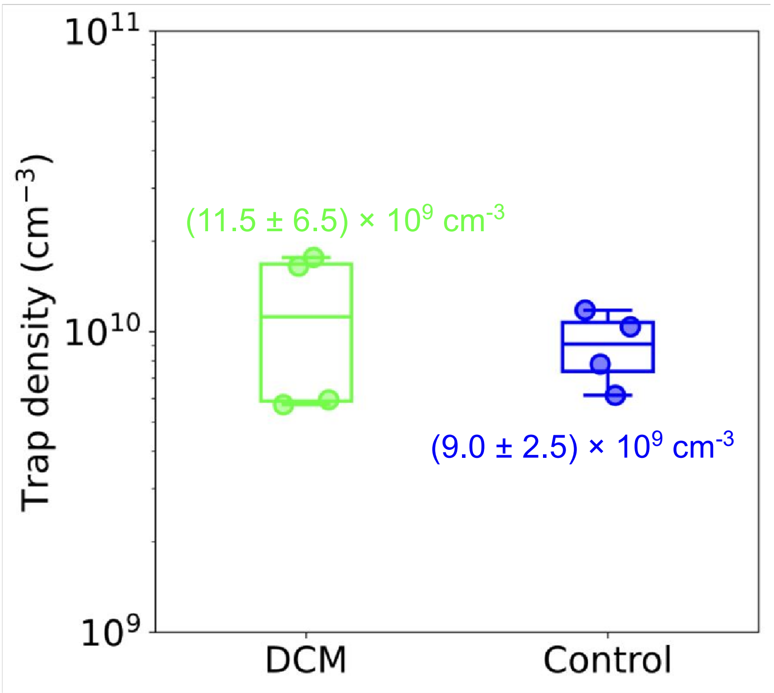

Figure S10. Trap density of DCM-0.2M and Control-1M crystals.

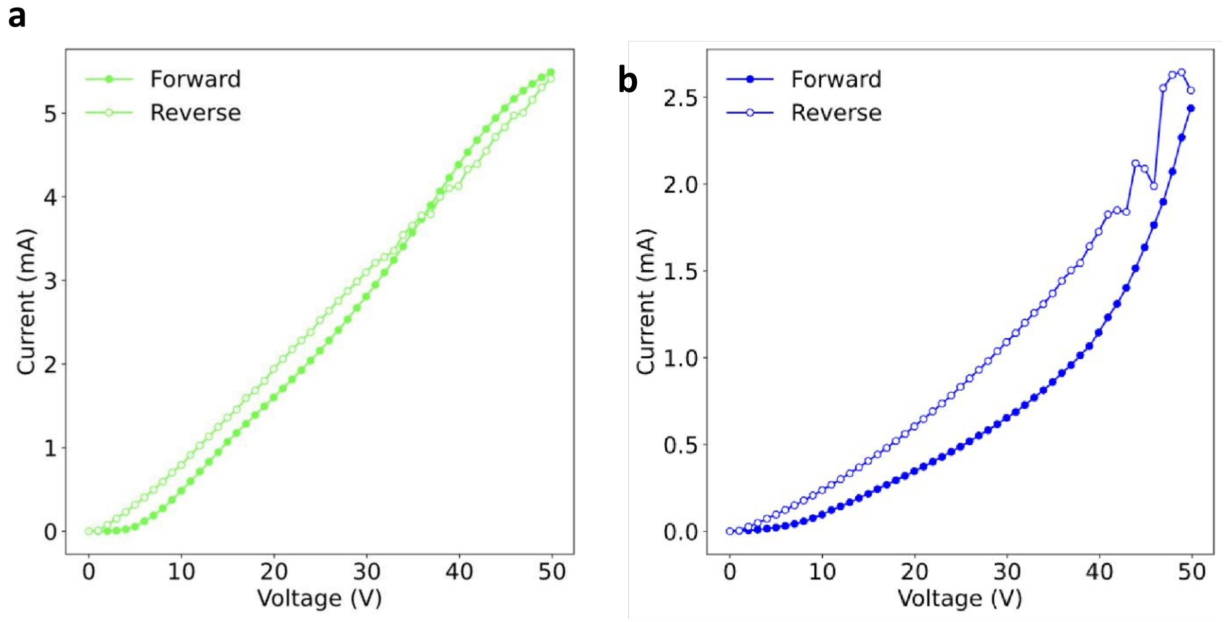

Figure S11. Hysteresis in the *I-V* curves of the devices. (a) DCM-0.2M (b) Control-1M. Continuous voltages were applied from 0 V to 50 V (forward scan) and from 50 V to 0 V (reverse scan) with a scan rate of 2 V/s. Both crystals have the same thickness of 1.3 mm.

(Page 14: Experimental section)

Pulsed-Voltage Space-Charge Limited Current measurements. The devices for PV-SCLC measurements were prepared by depositing carbon paste on each surface of the $MAPbBr_3$ crystals.[65] Then, pulsed voltages of 0.05–200 V were applied to the devices with the ON-time of 20 ms and the interval time of 120 s. Based on the log*I*-log*V* plots, we determined the onset voltage of the trap-filled limit regime (V_{TFL}) as shown in Figures S8 and S9. The trap density (n_{trap}) was calculated by using the following equation:

$$n_{trap} = \frac{2V_{TFL}\varepsilon\varepsilon_0}{eL_2}$$

where ε_0 represents the vacuum permittivity (8.85 × 10^{-12} F m^{-1}), ε: the relative dielectric constant of $MAPbBr_3$ (25.5),[4] *e*: the charge of the electron (1.6 × 10^{-19} C), *L*: the thickness of the crystal (mm).

5. A comparison Table of photodetection properties of X-rays detectors should be made for the reported in this draft and reported by others lead halide perovskite SCs based photodetectors.

Our Response:

We now provide a comparison in Table S1 in supporting information. See Page 11 in the revised manuscript and Table S1.

The obtained sensitivity is compared with the reported perovskite X-ray detectors (Table SI). Our detectors showed a high sensitivity among them even though the electric field is not high.

Table SI. Comparison of direct-conversion perovskite X-ray detectors.

Material	Thickness (mm)	Device structure	kVp	Electric field (V mm^{-1})	Sensitivity (μC Gy_{air-1} cm^{-2})	Ref
$MAPbBr_3$ single crystal	2	Au/PVK/C_{60}/BCP/Ag or Au	-	-	80	1
$MAPbI_3$ film	0.83	ITO/PI PVK/PVK/PI PVK/TFT/ITO	100	240	11,000	2
$MAPbBr_{2.94}Cl_{0.06}$ single crystal	1	Cr/C_{60}/BCP/PVK/Cr	8	60	84,000	3
$MAPbBr_3$ single crystal	2	Si/PVK/C_{60}/BCP/Au	50	0.5	322	4
$MAPbI_3$ wafer	1	Ag/ZnO/PCBM/PVK/PEDOT:PSS /ITO	70	200	2527	5
$MAPbBr_3$ single crystal	7	Au/poly-TPD/PVK/C_{60}/PCBM/Ag	30	14.3	23,600	6
$CsPbBr_3$ film	0.24	FTO/PVK/Au	50	5	55,684	7
$CsPbBr_3$ film	0.018	Au/PVK/Pt	35	110	1,700	8
$MAPbBr_3$ single crystal	2	AZO/PVK/Au	80	5	529	9
$MAPbBr_3$ single crystal	2.6	Al/PVK/Au	50	14300	359	10

$MAPbBr_3$ single crystal	3	Au/PVK/Au	39	0.83	259.9	11
$MAPbBr_3$ single crystal	-	Cr/BCP/C60/PVK/Au	120	-	3,928.3	12
$MAPbI_3$ wafer	0.8	Au/PCBM/PVK/Au	40	12.5	122,000	13
$MAPbBr_3$ single crystal	2.02	InGa/C_{70}/PVK/Au	-	1.98	184.6	14
$MAPb(I_{0.9}Cl_{0.1})_3$filled membrane	0.24	Cr/BCP/C_{60}/PVK/Cr	50	50	2,204	15
$Cs_2AgBiBr_6$ film	0.092	W/PVK/Pt	70	109	487	16
$MAPbBr_3$ film/$MAPbBr_3$ single crystal	2	Au/PVK/Ga	40	5	549.9	This work

6. Passivation of SC surface may have a significant effect on ion migration. Is it possible to study the impact of the proposed passivation on the activation energy of MAPbBr3 SC?

Our Response: As discussed in above, we observed less hysteresis in the DCM-0.2M device compared to the Control-1.0M. While this could indicate reduced ion migration in the DCM-0.2M crystals, it could also suggest less charge accumulation at the interface between perovskite and electrodes.

7. Figure 3b shows only a dark I-V plot of both devices. Please provide the relevant I-V plot of both devices under 25.2 Gyair s-1.

Our Response: We measured the *J*-*V* plot of both devices under 25.2 μGy_{air} s^{-1} and noticed that the *J*-*V* plots are not reproducible probably due to ion migration occurring in both crystals (Figure R3). Since the voltage sweep from -40 V to 40 V gives a high electric field in the crystal, the dark current and photocurrent were always different each time we measured them. Therefore, the difference between the photocurrent and the dark current in the *J*-*V* plots would not be the response current. To avoid this misleading, we would like to keep the Figure 3b as it is. We believe measuring the ON/OFF current at a constant bias such as Figure 3c and Figure S13 (in the reply to Comment 9) is more proper to evaluate the response current. Meanwhile, the leakage current at the negative bias was always higher in the Control devices, still suggesting the charge recombination at the interface due to defects.

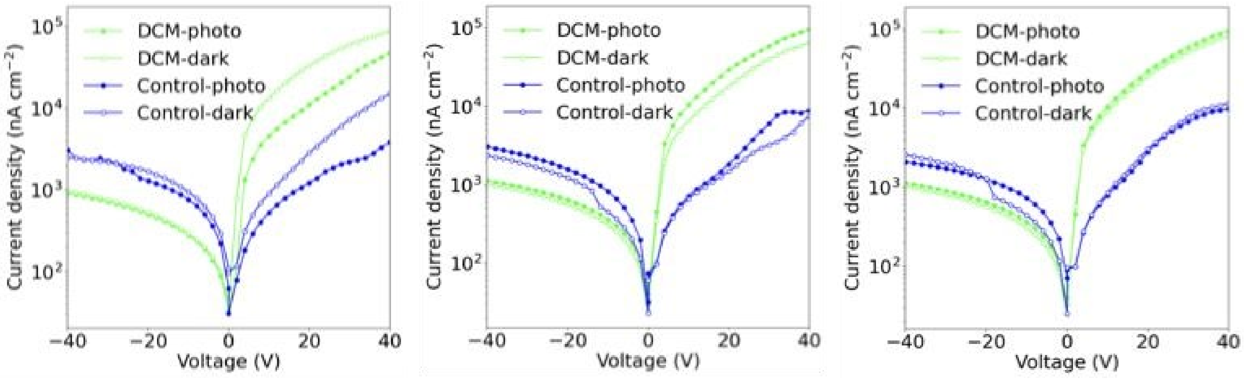

Figure R3. Three *J-V* plots of the devices under dark and X-ray (25.2 μGy/s) irradiation from the same device (DCM and Control).

8. There is no information related to Au electrode thickness. For X-ray measurement, the thickness of the top electrode is essential.

Our Response: The Au thickness is 80 nm which should not attenuate the X-ray too much. We now added this information in the method section.

Au electrodes (80 nm in thickness) were thermally evaporated on the surface of the as-deposited $MAPbBr_3$ single crystals.

9. In principle, X-ray detectors are working under applied high bias. Why the authors did not test the device under high 40 V?

Our Response: We measured the response under -20 V bias.

We have included the text and figure below in the revised version. See Page 12 in the revised manuscript and Figure S13.

We also measured the response current of the DCM-0.2M device at the higher bias of -20 V (Figure S13). Although the DCM-0.2M device showed higher response currents leading to the sensitivity of 898.8 μC Gy_{air}^{-1} cm^{-2}, the current was unstable same as the Control-1M at the bias of -5 V (Figure 3c). The SNR for 25.2 μGy_{air} s^{-1} X-ray was 36.2, which is less than the SNR of 117.6 when the bias was -5V.

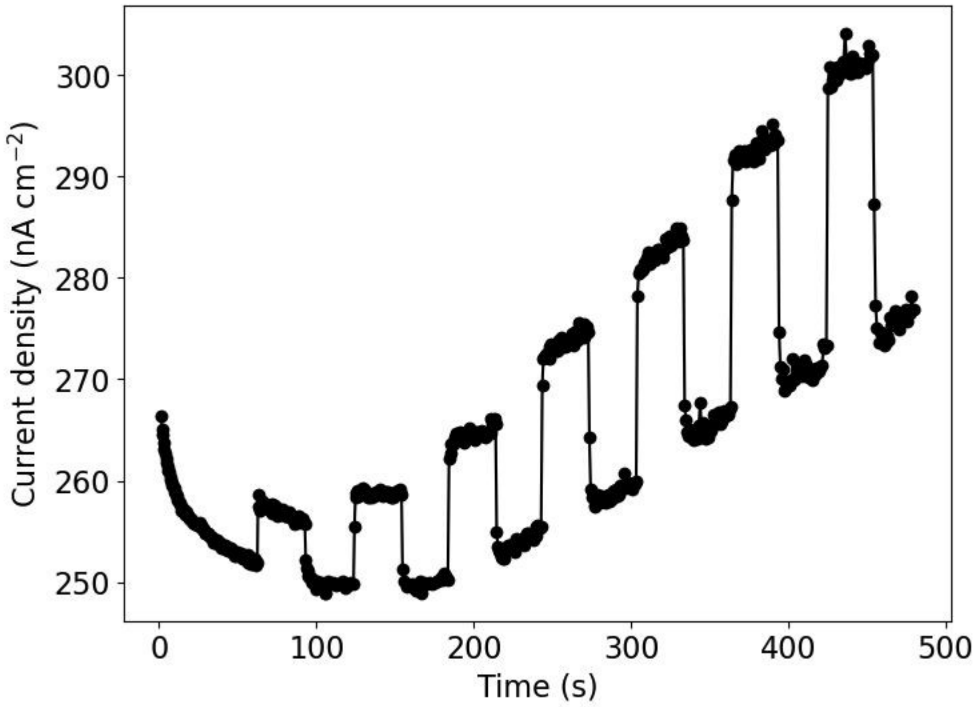

Figure S13. The response current of the DCM-0.2M device at the bias of -20V. The X-ray dose rates are the same as the ones in Figure 3c.

10. Some relevant literature works on the passivation of MAPbBr3 SC (Journal of Applied Physics 127, 185501 (2020)) and MAPbBr3 SC based photodetectors (ACS Photonics 2023, 10, 1424) are missing.

Our Response: The relevant references are now cited in the revised version. See references 19 and 25.

Reviewer: 3

Recommendation: While the work is good and publishable, a more appropriate journal is recommended such as Crystal Growth & Design

Comments:

The authors provided alternative synthetic strategy to improve the MAPbBr3 single crystal with effective surface passivation to demonstrate overall quality of the single crystal, by presenting the intense green UV light, elongated PL decay lifetime, lower defect density with 5 times higher Xray detecting ablity. Through XPS, TRPL and dark J-V curves, the authors presented their crystals have better crystalline quality by decreasing the surface defect density. However, considering the manuscript scope, the description towards to demonstrate higher crystalline quality for better Xray detecting ability, it seems like the manuscript would be re-considered where to be published in the Journal of Physical Chemistry Letters due to mismatch journal aim and the flow/purpose of this manuscript. Overall supporting characterizations could support the enhanced quality of the crystal, so this manuscript could be considered to more suitable aim, such as Crystal Growth & Design, or any of crystal focused journals.

Our Response: We thank the reviewer for their comments. However, we would like to disagree regarding the mismatch of our work with the journal aim. Journal of Physical Chemistry Letters Subject Categories include Physical Insights into Materials and Molecular Properties, Physical Insights into Light Interacting with Matter, and Physical Insights into Chemistry, Catalysis, and Interfaces. Our work presented in this manuscript does fall under these categories. Furthermore, the Journal of Physical Chemistry Letters has previously published several relevant works on the growth of perovskite single crystals for X-ray detectors such as Zhang et al. *J. Phys. Chem. Lett.* 2020, 11, 2, 432–437, Xu et al *J. Phys. Chem. Lett.* 2021, 12, 1, 287–293, and Song et al *J. Phys. Chem. Lett.* 2020, 11, 9, 3529–3535. Therefore, we reckon that our manuscript matches the aim of the Journal of Physical Chemistry Letters.

Also, the authors could be consider the following aspects for the manuscript, as follows:

1. In page 8, line 56, there is no Figure2e in the manuscript. The authors should check again about the description and/or typo.

Our Response: Figure 2e is already included at the bottom of Figure 2c. However, thanks to the reviewer, we noticed that in Figure 2 caption, (d) was repeated twice. It is now corrected in the revised version.

2. Like as the experiment shown in Figure 3a and 3b, the authors may provide defect density differences through Space Charge Limited Current (SCLC) method. To demonstrate the enhanced crystalline quality as well as decreasing the defect density in both surface and inside of the crystal, more detailed analysis would need to be proposed.

Our Response: We measured the trap density of both crystals using four crystals of each. Both crystals showed similar trap densities. We think this is because SCLC gives bulk property rather than surface property. However, PL results still suggest the DCM-0.2M crystals have fewer defects at the surface. Furthermore, we observed less hysteresis in the DCM-0.2M device compared to the Control-1.0M, suggesting less charge accumulation at the interface between perovskite and electrodes, which means a better surface of the DCM-0.2M crystal. These results suggest that DCM-0.2M and Control-1M have similar trap densities in the bulk however; DCM0.2M has less defect at the surface. Thus, we may conclude that the surface properties significantly affect the performance of the X-ray detectors using perovskite single crystals even if the bulk properties are similar, in agreement with the previous research reporting the improvement in the device performance by post-surface treatment (Chen et al. *ACS Appl. Mater. Interfaces* **2022,** *14*, 10917-10926).

We have included the text and figure below in the revised version. See Page 10 in the revised manuscript and Figure S8-11.

The trap densities of both crystals were measured by using 4 crystals of each with the pulsedvoltage space-charge limited current (PV-SCLC) method (Figures S8 and S9).[61] As shown in Figure S10, both crystals showed similar trap densities (DCM-0.2M: 11.5×10^9 cm^{-3}, Control-1M: 9.0×10^9 cm^{-3} on average). The trap density obtained by PV-SCLC reflects the bulk property rather than the surface property. On the other hand, when we applied continuous voltages to the devices, we observed less hysteresis in the DCM-0.2M device compared to the Control-1.0M (Figure S11), suggesting less charge accumulation at the interface between perovskite and electrodes, which indicates a better surface of the DCM-0.2M crystal. These results suggest that DCM-0.2M and Control-1M have similar trap densities in the bulk however; DCM-0.2M has less defect at the surface.

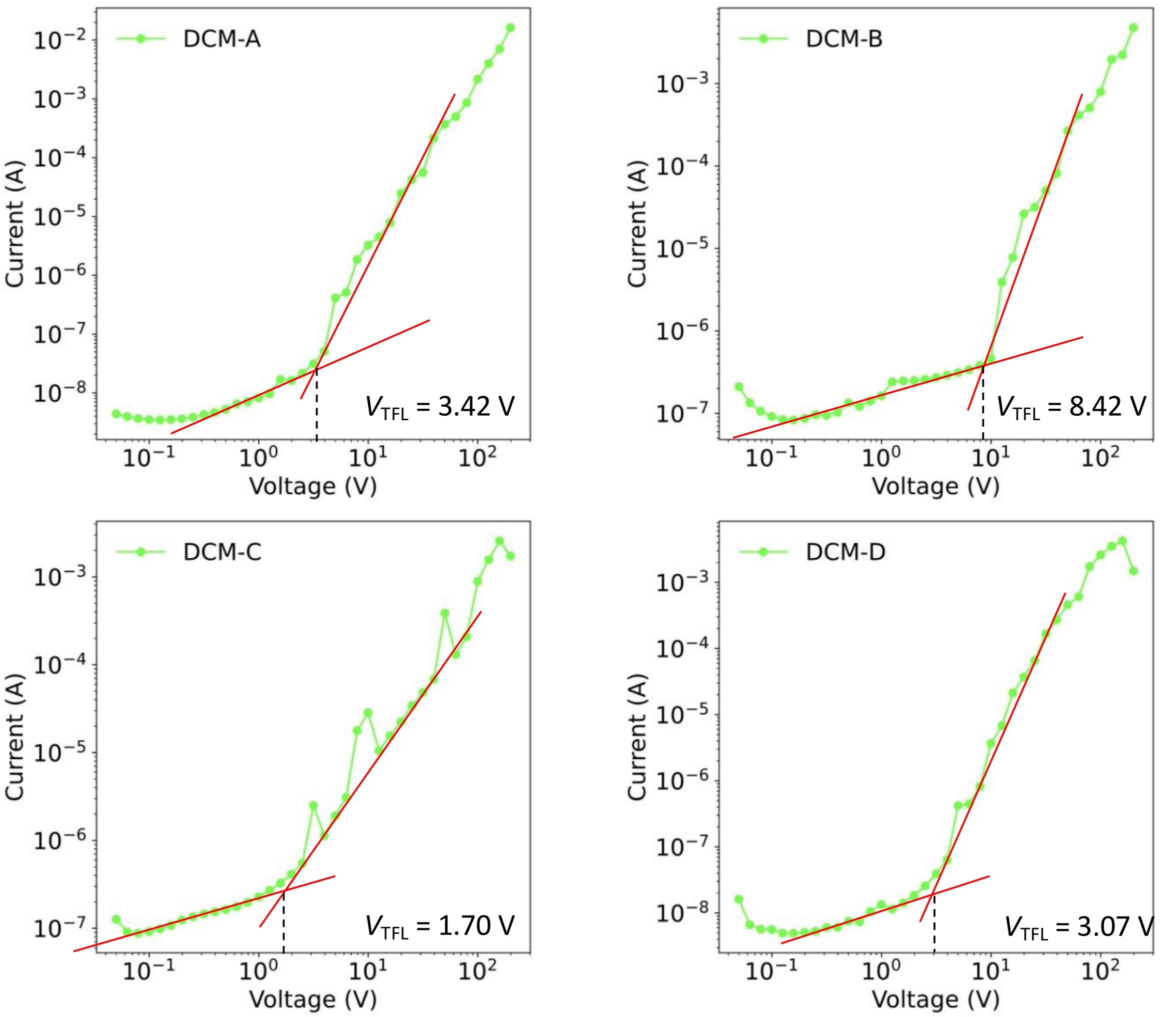

Figure S8. The log*I*-log*V* plots of DCM-0.2M crystals.

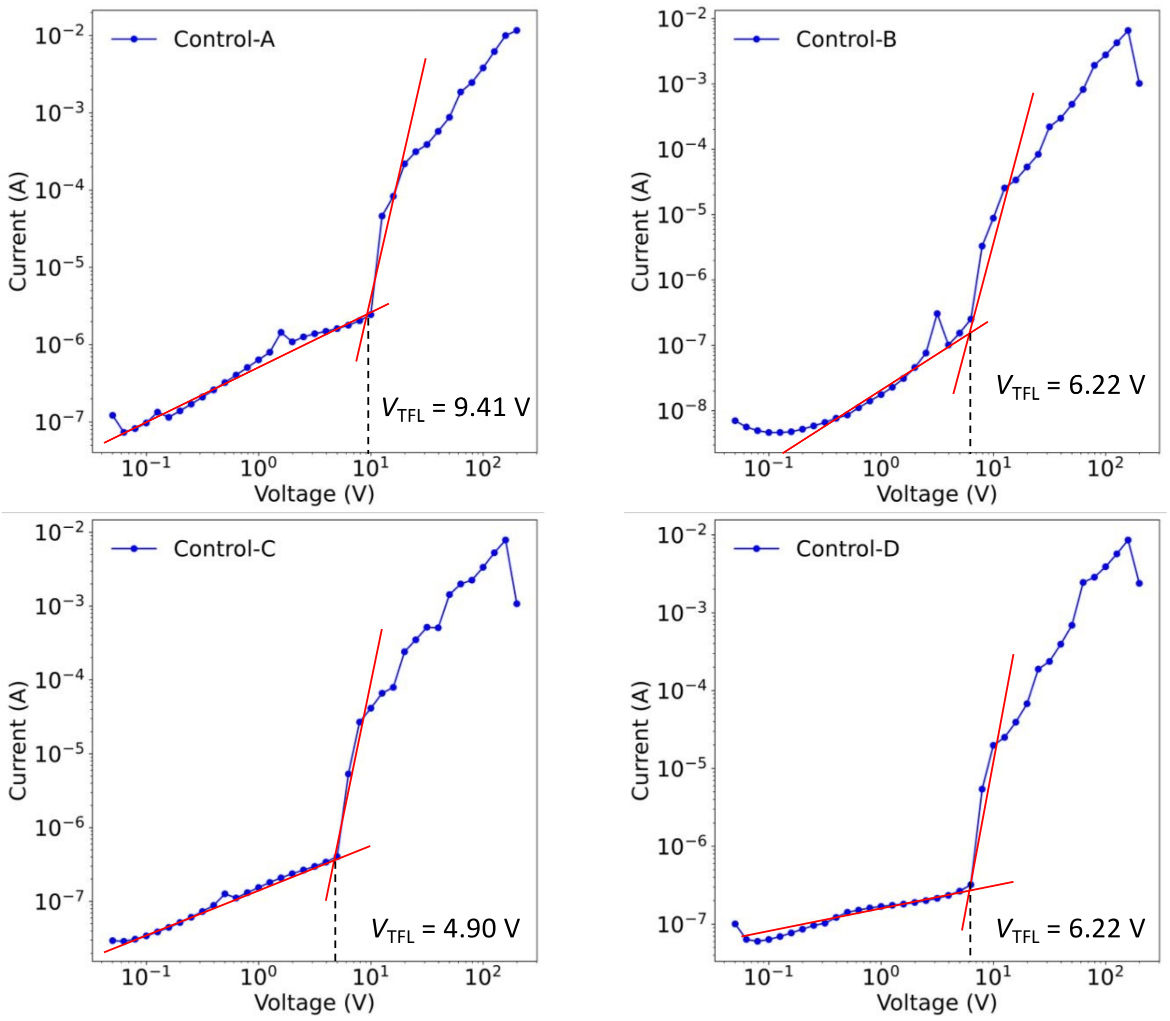

Figure S9. The log$I$-log$V$ plots of Control-1M crystals.

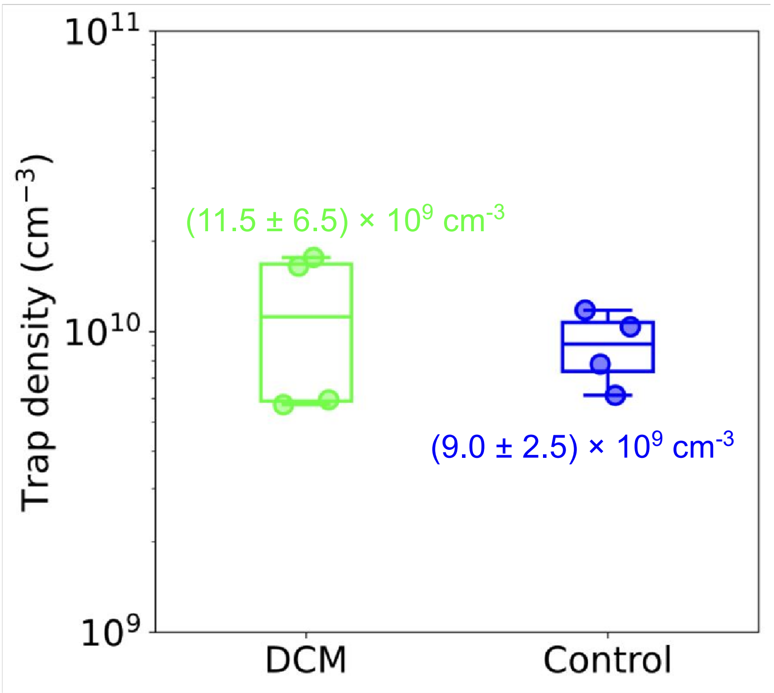

Figure S10. Trap density of DCM-0.2M and Control-1M crystals.

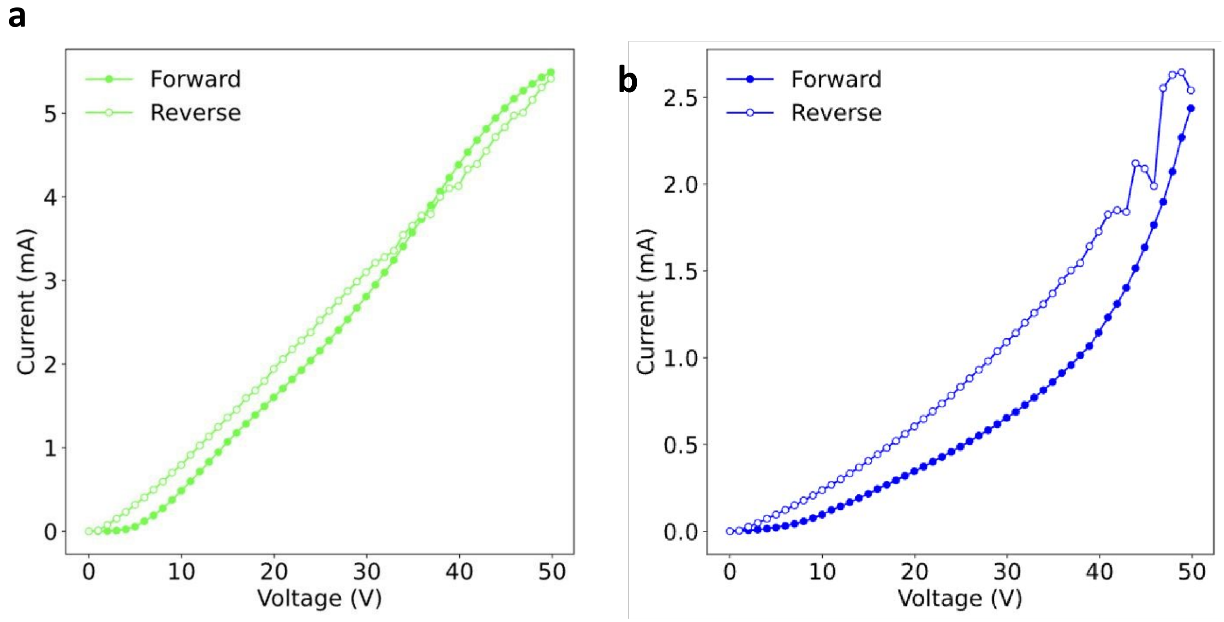

Figure S11. Hysteresis in the *I-V* curves of the devices. (a) DCM-0.2M (b) Control-1M. Continuous voltages were applied from 0 V to 50 V (forward scan) and from 50 V to 0 V (reverse scan) with a scan rate of 2 V/s. Both crystals have the same thickness of 1.3 mm.

(Page 14: Experimental section)

Pulsed-Voltage Space-Charge Limited Current measurements. The devices for PV-SCLC measurements were prepared by depositing carbon paste on each surface of the $MAPbBr_3$ crystals.[65] Then, pulsed voltages of 0.05–200 V were applied to the devices with the ON-time of 20 ms and the interval time of 120 s. Based on the log*I*-log*V* plots, we determined the onset voltage of the trap-filled limit regime (V_{TFL}) as shown in Figures S8 and S9. The trap density (n_{trap}) was calculated by using the following equation:

$$n_{trap} = \frac{2V_{TFL}\varepsilon\varepsilon 0}{eL_2}$$

where ε_0 represents the vacuum permittivity (8.85×10^{-12} F m^{-1}), ε: the relative dielectric constant of $MAPbBr_3$ (25.5),[4] *e*: the charge of the electron (1.6×10^{-19} C), *L*: the thickness of the crystal (mm).
